# SimUrine: a novel, fully defined artificial urinary medium for enhanced microbiological research of urinary bacteria

**DOI:** 10.1128/aem.01559-25

**Published:** 2026-01-09

**Authors:** Pablo Gallardo Molina, Brian I. Choi, Michael Vanek, Mohammad Haneef Khan, Kathrin Tomasek, Ayla N. Kwant, Peter Dijkstra, Marjon G. J. de Vos, Alan J. Wolfe

**Affiliations:** 1 Department of Microbiology and Immunology, Loyola University Chicago, Maywood, Illinois, USA; 2 GELIFES-University of Groningen 195329 , Groningen, the Netherlands; 3 Laboratory of Microbiology and Microtechnology, School of Life Sciences (SV), Ecole Polytechnique Fédérale de Lausanne (EPFL) 27218, Lausanne, Switzerland; 4 Polymer Science, Zernike Institute for Advanced Materials, University of Groningen 3647, Groningen, the Netherlands; Centers for Disease Control and Prevention, Atlanta, Georgia, USA

**Keywords:** artificial urine, urobiome, urinary tract infection, microbiome research, bacterial cultivation, microbial ecology

## Abstract

Urinary tract infections represent one of the most prevalent bacterial diseases, yet current diagnostic and research methodologies are hampered by inadequate culture media that fail to replicate the bladder biochemical environment. Conventional artificial urine formulations contain undefined components, lack essential nutrients, or inadequately support urinary microbiome (urobiome) growth. To address these limitations, we developed SimUrine, a fully defined synthetic urine medium that aims to replicate human bladder chemistry while supporting diverse microbial growth requirements. SimUrine was systematically developed through iterative optimization of multi-purpose artificial urine, incorporating defined concentrations of carbon sources, vitamins, trace elements, and amino acids within physiologically relevant ranges. The modular design enables component substitution without complete reformulation, facilitating customization for culturomics, antimicrobial susceptibility testing, and microbial ecology studies, while reducing batch-to-batch variability associated with authentic urine. Performance evaluation demonstrated SimUrine's capability to support the growth of fastidious urobiome members, including *Lactobacillus* species, *Aerococcus urinae*, and *Corynebacterium riegelii*, which fail to proliferate in conventional minimal media. Physicochemical characterization confirmed that SimUrine formulation exhibits properties within normal human urine ranges for density, conductivity, osmolarity, and viscosity, ensuring physiological relevance. Clinical applications revealed reduced antibiotic susceptibility compared to standard media, suggesting a more accurate representation of *in vivo* conditions. Co-culture experiments using *Escherichia coli* and *Enterococcus faecalis* demonstrated previously unobserved microbial interactions, highlighting SimUrine's utility for investigating urobiome dynamics. SimUrine represents a significant advancement in urobiome research methodology, providing a standardized, reproducible platform for investigating the urobiome under physiologically relevant conditions, potentially improving fundamental understanding and clinical diagnostic approaches.

**IMPORTANCE:**

Urinary tract infections (UTIs) affect millions globally, yet current research and diagnostic methods rely on inadequate culture media that fail to replicate the bladder's unique biochemical environment. This fundamental limitation has hindered accurate UTI research and potentially compromised clinical treatment decisions. SimUrine addresses this critical gap as the first fully defined synthetic urine medium that mimics human bladder chemistry while supporting the growth of diverse urinary microbes. The breakthrough enables the cultivation of urobiome organisms in a minimal medium that resembles natural conditions, revealing novel microbial interactions that influence urinary health. Crucially, SimUrine demonstrates different antimicrobial susceptibility patterns compared to standard clinical media, suggesting current testing protocols may inaccurately predict treatment outcomes. This standardized, reproducible platform eliminates the variability of authentic urine samples while maintaining physiological relevance, potentially transforming urobiome research methodology and providing a new tool for the study of UTIs worldwide.

## INTRODUCTION

Urinary tract infections (UTIs) are among the most common bacterial diseases worldwide. They are particularly common in females; approximately 50%–60% will experience at least one UTI in their lifetime (1). The diagnosis of UTIs can be challenging due to the variable presentation of symptoms, including pyuria, dysuria, and fever, which are not present in all cases. Symptom inconsistency has provoked debates within the medical community regarding standardized diagnostic criteria (2). Another significant challenge in UTI diagnosis is the discrepancy between these symptoms and the bacterial counts enumerated from standard urine culture technique, a methodology that often produces false negative results, particularly overlooking fastidious and anaerobic bacteria (3, 4). Newer diagnostic techniques with heightened sensitivity have increased the detection rates of bacteria among symptomatic individuals (3, 5, 6). However, our understanding of these detected bacteria is limited, especially concerning the role of hard-to-cultivate species of the urinary microbiome (urobiome) in the context of UTIs (4, 6).

These limitations stem partly from the lack of culture media that accurately replicate the bladder’s metabolic environment. Commercially available media, fortified with general nutrients, fail to replicate the nutritional composition and complexity of human urine (7). Using authentic urine samples introduces significant donor-to-donor variability, compromising experimental reproducibility (8, 9). Another strategy is to use pooled urine, which has been theorized to reduce variation. However, a recent report showed that pooled urine is artificially optimized and not average (10). Thus, without a standardized urine medium, comparing experimental results between experiments and different laboratories remains difficult.

Unfortunately, current formulations aiming to replicate bladder-like conditions either contain undefined components, do not encompass the complete range of essential nutrients, or are inadequate to support the growth of the broad spectrum of urinary microbes (11, 12). Salt-based medium formulations have been developed to mimic the electrolyte composition of urine; nevertheless, these fail to sustain microbial growth due to their lack of or limited carbon content and absence of other vital nutrients (13, 14). To achieve a standardized synthetic urine medium, it is essential to recreate the conditions that members of the urobiome encounter in their natural habitat (9), incorporating carbon sources and vital nutrients necessary for the growth of diverse urinary microbes, including species considered to be pathogenic or commensal.

The objective of this study is to develop a fully defined medium that accurately replicates the chemical composition of urine in the human bladder and accommodates various experimental applications, including culturomics, co-evolutionary research, and synthetic communities. To achieve this end, we modified multi-purpose artificial urine (MP-AU) (14), incorporating defined concentrations of carbon sources, vitamins, and salts, with the aim to maintain medium concentrations within the ranges reported for human urine. To determine the efficacy of our formulations, the experimental media were tested for their ability to support the growth of a diverse set of target microbes. To show possible applications of our medium, we have included co-culture and antibiotic resistance experiments.

The resultant medium, termed SimUrine, alleviates the inconsistencies associated with the use of actual urine and ensures more uniform outcomes in urobiome investigations. SimUrine also exhibits physicochemical properties within the normal range of human urine, including density, conductivity, refractive index, and viscosity, making for a physiologically relevant *in vitro* artificial medium.

SimUrine’s composition is structured modularly, allowing for the substitution of individual components to accommodate varying research requirements without requiring full medium reconstruction. The modular design also enables facile, prolonged storage and replacement of components upon their expiration date. SimUrine supports a wider range of species than conventional minimal media while sustaining antibiotic susceptibility testing and displaying comparable or enhanced microbial survival rates, including for many fastidious urobiome members.

In summary, SimUrine addresses the significant constraints of existing artificial urine media by providing a fully defined, customizable, nutrient-optimized medium for diverse research applications, enhancing our comprehension of urinary microbial viability and interactions in closely replicated bladder environments.

## MATERIALS AND METHODS

### Bacterial strains

Urinary bacterial isolates used in this study were sourced from strain collections of the Wolfe lab collection at Loyola University Chicago or the de Vos lab at the University of Groningen (Table S1).

### Reference media

Our initial experiments utilized NYCIII medium (15) as either a reference or to start cultures (as indicated in each case), due to the fastidious growth requirements of *Lactobacillus* species. However, to ensure simplicity and reproducibility, we conducted subsequent experiments using brain heart infusion (BHI) medium, except for the antibiotic susceptibility testing, for which Mueller-Hinton broth (MHB) was used to culture reference strains.

### Optimization of MP-AU for bacterial growth

To assess bacterial viability in MP-AU (14), we evaluated *Escherichia coli* (UMB1180) growth and survival in a modified version (mMP-AU), where K_2_C_2_O_4_ · H_2_O was replaced by NH_4_C_2_O_4_ · H_2_O. For this aim, we first grew the bacteria in NYCIII medium (15) for 48 hours at 37°C in 5% CO_2_ and diluted to an optical density at 600 nm (OD_600_) = 1. The culture was centrifuged, washed three times using PBS 1×, and resuspended in either 1 mL of mMP-AU or M9 salts. A 500 µL aliquot of this resuspension was inoculated into 5 mL of mMP-AU or M9 salts and incubated at 37°C for 4 days in 5% CO_2_ with continuous orbital agitation at 200 rpm. Samples were taken every day, and serial dilutions were plated on BHI agar for enumeration of colony-forming units (CFU/mL) to determine survival.

To determine if an enriched version of mMP-AU could support the growth of fastidious species, we tested urinary isolates of *Lactobacillus jensenii* (UMB8651) and *Aerococcus urinae* (UMB5254, aka type strain ATCC 51268) in mMP-AU supplemented with various glucose concentrations. To evaluate additional nutritional requirements, mMP-AU was fortified with an equimolar carbon source (1% each) mix (glucose, galactose, maltose, sucrose, fructose, mannitol, and sorbitol), a vitamin mixture (thiamin, 5.0 mg/L; riboflavin, 5.0 mg/L; niacin, 5 mg/L; pantothenic acid, 5.0 mg/L; pyridoxine hydrochloride, 10.0 mg/L; biotin, 2.0 mg/L; and 4-aminobenzoic acid, 5.0 mg/L) and casamino acids. Bacterial cultures were evaluated daily for survival, enumerating CFU/mL on Columbia colistin nalidixic acid agar plates under 5% CO_2_.

To assess the impact of residual nutrients from rich media on bacterial growth in nutrient-limited conditions, strains of *E. coli* (UMB3190, aka ATCC 10798) and *Klebsiella pneumoniae* (UMB9987) were first grown overnight in BHI. These precultures were then diluted 1:100 into either mMP-AU or M9 salts and cultured under our standard conditions: 48 hours at 5% CO_2_, 37°C with orbital agitation of 200 rpm. Growth was monitored in a 96-well clear flat bottom plate by measuring OD_600_ using a Biotek reader (Winooski, VT, USA). These were single exploratory experiments; no replicates were done.

### SimUrine formulation optimization

Several versions of SimUrine were tested until we were able to grow a wider group of organisms and produce a stable medium; the final formulation (SimUrine.v6) is described in Protocol S1 and Table S2. Other versions are included for reference in Protocol S2. By making these different SimUrine formulations, we aimed to mimic the nutrient-limited bladder environment and maximize the number of organisms able to grow in our formulation, through progressive enrichment of mMP-AU and by tuning reagent concentrations to stay within urine ranges, while designing a modular protocol to enable facility, prolonged storage, and easy component replacement. Each formulation was tested for growth of specific isolates, previously cultured on BHI agar plates.

The first version of SimUrine (SimUrine.v1) was formulated as follows: mMP-AU supplemented by adding N-acetylglucosamine, L-threonine, and L-serine (to mimic mucin composition without the turbidity or contamination problems associated with direct use of mucins) and lactic acid, pyruvic acid, acetic acid, manganese sulfate, and L-cysteine (to protect facultative species and improve fastidious species growth). Bacterial growth of common urobiome organisms was assessed for two consecutive periods of 48 hours.

Based on the performance of SimUrine.v1, and with the aim to extend use of the medium to a wider range of microbes and increase growth, a second version of SimUrine was designed (SimUrine.v2). Inspired by the work on bacterial communities from the gut (16), we incorporated vitamins and trace elements into this formulation.

Given the biological relevance of iron and aiming to increase the number of species able to grow in SimUrine, we assessed the impact of hemin supplementation in our formulation with two concentrations: one as described in SimUrine.v3 and one 10-fold greater. Thinking of the stability of our formulation and possible stress on bacterial isolates, we evaluated the need for uric acid in our medium by growing strains in the presence and absence of this compound.

To reduce spontaneous precipitate formation, improve medium stability, and keep uric acid in our formulation, the reagent order was modified in SimUrine.v4: now, uric acid was added after urea and not before. We also improved buffering capacity of the medium with the addition of 4-(2-hydroxyethyl)-1-piperazineethanesulfonic acid (HEPES), and to better mimic the nutrient profile of urine, we added non-ionic surfactant Tween 80 and extra peptides (7, 17). Given the role of cysteine as a key energy and carbon source in various environments (18), we also assessed the use of its dimeric form, cystine, by testing a version of SimUrine.v4 with cysteine replaced by cystine. With this, we expected to fit the requirements for other organisms that had not yet grown in previous formulations, while keeping the growth of those being successful.

Aiming to extend the lifespan of our formulation, minimize precipitate formation during storage, and reduce chelation of trace elements, we incorporated sodium bicarbonate and 3-(N-morpholino) propanesulfonic acid (MOPS) as buffering agents instead of HEPES in SimUrine.v4, resulting in SimUrine.v5. In contrast to earlier formulations that included autoclaving of the final medium, this new formulation was sterilized by filtration with a 0.2 µm filter.

Considering the limited number of organisms able to grow in our different formulations, we optimized SimUrine.v5 with reagents expected to be essential for optimal growth of *Enterococcus faecalis*, *Lactobacillus*, and *Proteus*; for this aim, we incorporated glucose, FeSO_4_, L-valine, L-threonine, and L-tryptophan (19–23). Additionally, pH was reduced from 6.5 to 6.0 to increase stability. Three different concentrations of urea were evaluated at this stage. Based on these experiments, a final version, including these compounds and urea (15 g/L), was adopted as SimUrine.v6. A version of SimUrine.v6 buffered at pH = 5.0 was used to provide acidic conditions similar to those of the vagina, and to confirm that our formulation could support *Lactobacillus* growth.

### SimUrine evaluation

To assess the performance of the different SimUrine versions, pure bacterial isolates were first grown overnight in a 20 mL glass tube containing 4 mL of the corresponding version of SimUrine at standard conditions (37°C, 5% CO_2_ with orbital agitation at 200 rpm). Overnight cultures were diluted 1:100 into the noted SimUrine versions, and growth was monitored in 96-well clear flat-bottom plates under standard conditions (37°C, 5% CO_2_, and orbital agitation at 200 rpm) for 24, 48, or 72 hours (depending on the species). OD_600_ was assessed every 10 minutes using Biotek (Winooski, VT, USA) or Cerillo Alto (Charlottesville, VA, USA) plate readers (indicated with each result). OD_600_ values for control wells containing SimUrine are indicated in each figure legend. To confirm species identity, colonies were obtained from the cultures at the end of each growth curve and identified using mass spectrometry on a Bruker MALDI-TOF (Billerica, MA, USA). Formulations were continually modified aiming to allow the growth of all listed organisms (Table S1) and improve stability of the medium. The initial set of bacteria (for SimUrine.v1) was selected as expected to grow with minimal nutritional requirements; more fastidious bacteria were evaluated as the complexity of the formulation increased.

### Physicochemical properties of SimUrine.v6

To characterize standard SimUrine.v6 for broader applications, physicochemical parameters (density, refractive index, viscosity, conductivity, osmolarity, and absorbance) were determined from two independent batches prepared 2 weeks apart, each measured in technical triplicate at 20°C. Density was determined using a volumetric flask and an analytical balance, refractive index was measured using a KERN Abbe ORT 1RS refractometer (Kern & Sohn, Balingen, Germany), viscosity using an MCR 702e rheometer (Anton Paar, Breda, The Netherlands), conductivity using a portable conductivity meter (Mettler-Toledo, Columbus, OH, USA), osmolarity using an Omo1 osmometer (Advance instruments, Norwood, MA, USA), and absorbance at OD_600_ using a Cerillo Alto reader (Cerillo, Charlottesville, VA, USA) at 20°C.

### *Lactobacillus* growth

To confirm the growth of *Lactobacillus* in SimUrine.v6, strains were plated in CHROMagar (Condalab, Madrid, Spain) and then colonies cultured for 48 hours in 7 mL of SimUrine.v6 at pH = 5 at 37°C, using 15 mL conical tubes. Anaerobic conditions were achieved by culturing the strains inside an anaerobic jar (BD GasPak EZ) and using anaerobic atmosphere generation bags (Thermo Scientific Oxoid AnaeroGen). Growth was confirmed by OD_600_ and plating. CFU/mL were counted at time zero (*T* = 0H) and after 48 hours (*T* = 48H) using urinary tract infection CHROMagar (Condalab, Madrid, Spain). The identity of colonies was confirmed by MALDI-TOF (Billerica, MA, USA).

### Evaluation of SimUrine.v6 in microbial ecology-relevant experiments

To compare the performance of SimUrine.v6 for urobiome-related assays, antibiotic susceptibility tests were performed on *E. coli* (UPEC20). A 1:19 ratio of trimethoprim (TMP) and sulfamethoxazole (SMX) was tested at a starting concentration of 32 µg/mL TMP and 608 µg/mL SMX, with serial twofold dilutions. Susceptibility to kanamycin was also tested, starting at 128 µg/mL. MHB was used as the reference medium. In all assays, the initial inoculum was ~2.5 × 10^5^ CFU/mL. OD_600_ was measured in a 96-well plate using a Cerillo Alto reader (Cerillo, Charlottesville, VA, USA) at 37°C and 5% CO_2_. The antibiotic minimal inhibitory concentration (MIC) of *E. coli* was determined as μg/mL (based on ≥90% growth inhibition at 18 hours), as derived from the kinetic growth curve analysis.

### Co-culture of *E. coli* and *E. faecalis* strains

Reference strains of *E. coli* (ATCC 25922) and *E. faecalis* (ATCC 29212) were obtained from the American Type Culture Collection (ATCC). Prior to experimental use, both strains were sub-cultured onto trypticase soy agar supplemented with 5% sheep blood and incubated at 37°C in a 5% CO_2_ atmosphere for bacterial propagation and viability assessment. Overnight cultures of both bacterial strains were prepared in NYCIII medium and SimUrine.v6 medium, with a minimum of five biological replicates per strain. Cultures were incubated at 37°C with orbital shaking at 200 rpm. Following overnight incubation, cultures were harvested by centrifugation at 3,000 × *g* for 10 minutes at room temperature, supernatants were discarded, and bacterial pellets were resuspended in fresh medium (NYCIII or SimUrine.v6, respectively). Bacterial cell density was standardized by OD_600_, with all cultures adjusted to an initial OD_600_ of 0.05. For growth kinetic measurements, 80 μL of standardized bacterial suspension was combined with 720 μL of the appropriate growth medium (NYCIII or SimUrine.v6) in individual wells of a dual-well system (Cerillo co-culture duet system, Charlottesville, VA, USA). Control wells contained 800 μL of sterile medium without bacterial inoculum.

Growth kinetics were monitored over 24 hours using a Cerillo Alto microplate reader with continuous orbital shaking at 200 rpm at 37°C. OD_600_ measurements were recorded at 30 minutes intervals, and final readings at 24 hours were used for subsequent data analysis. Growth curves and standard error calculations were performed using MATLAB-R2025a (24).

### SimUrine.v6 protocol for 500 mL of media (abbreviated)

#### Preparation

Follow this abbreviated protocol in a sterile environment using aseptic technique to ensure reproducibility. All stock solutions must be prepared and either filtered (0.2 µm) or autoclaved separately before making the medium (see Protocol S1 and Table S2 for details and reagents).

Once stock solutions are prepared and sterilized, dissolve the module reagents sequentially as indicated into 200 mL of dH_2_O, stirring at 40°C: starting with the mMP-AU module, followed by the Amino acids module, the Carbon sources module, and the Others module. While the medium is warm, add 500 µL of Tween 80, 1.05 g of MOPS, and 7.5 g of urea. Continue adding the stock solutions sequentially, check and adjust pH to 6 with HCl or NaOH, add freshly prepared uric acid solution, and finally L-serine, as indicated in the protocol. Check pH and adjust if necessary. Bring the volume to 500 mL with H_2_O. Filter-sterilize the media using a 0.2 µm filter. To avoid precipitation, avoid storage at temperatures lower than 4°C. To extend lifespan, protect from light.

## RESULTS

### Optimization of mMP-AU for bacterial survival

**Fig 1:**
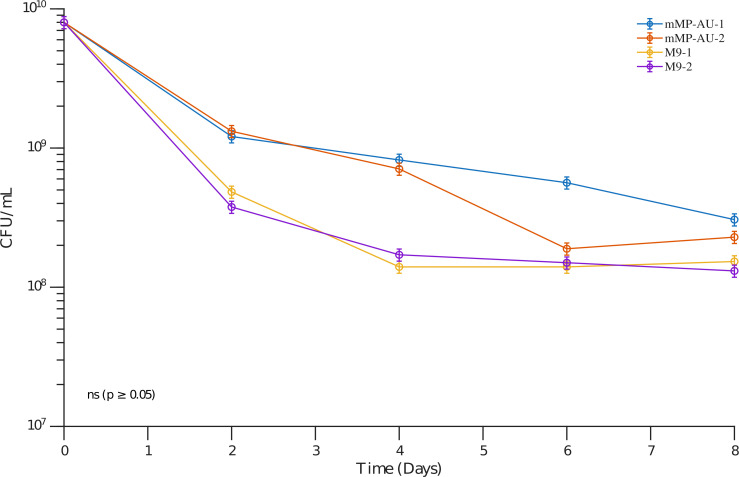
Survival of *E. coli* (UMB1180) in either mMP-AU or M9 salts. Two technical replicates were conducted for each condition, with each replicate averaging two biological replicates. The *y*-axis displays the logarithmic value of CFU/mL of *E. coli*, and the *x*-axis represents time in days. Standard deviation (SD) is displayed for each point. These results suggest that both salt bases similarly support the limited survival of urobiome species.

To compare the viability of *E. coli* (UMB1180) in minimal salt bases, we monitored survival over 8 days. Survival was marginally better in mMP-AU compared to M9 salts across the whole experiment (Fig. 1). This trend was maintained through day 8, indicating that mMP-AU supports bacterial viability at least as effectively as M9.

### Glucose supplementation influences survival of urinary isolates

To evaluate the contribution of a carbon source to the survival of urobiome species and aiming to begin the process of selecting the appropriate reagents to enrich mMP-AU, we incorporated glucose to the minimal salt bases. For mMP-AU and M9 salts, glucose supplementation had a contrasting effect on the survival of a urinary isolate of *L. jensenii* (UMB8651). For mMP-AU, a glucose concentration above 2% reduced survival, whereas elevated glucose concentrations had little effect on survival in M9 salts (Fig. S1A); however, survival on either salt base did not extend beyond 1 day. For *A. urinae* (UMB5254), increased glucose concentration was directly associated with enhanced survival when using mMP-AU as the salt base. In contrast to *L. jensenii* (UMB8651), *A. urinae* (UMB5254) survived for a second day, but only when using mMP-AU as the salt base. In contrast, when M9 was the salt base, no CFU of *A. urinae* were detected by day 1 (Fig. S1B).

As elevated glucose concentrations reduced *L. jensenii* (UMB8651) survival in mMP-AU, we tested the impact of various carbon sources and nutrients on UMB8651 growth in modified versions of mMP-AU. Growth was primarily supported by amino acids and vitamins, while other supplements failed to fully sustain bacterial proliferation (Fig. S2).

### Effect of residual nutrients from the inoculum on bacterial growth

**Fig 2:**
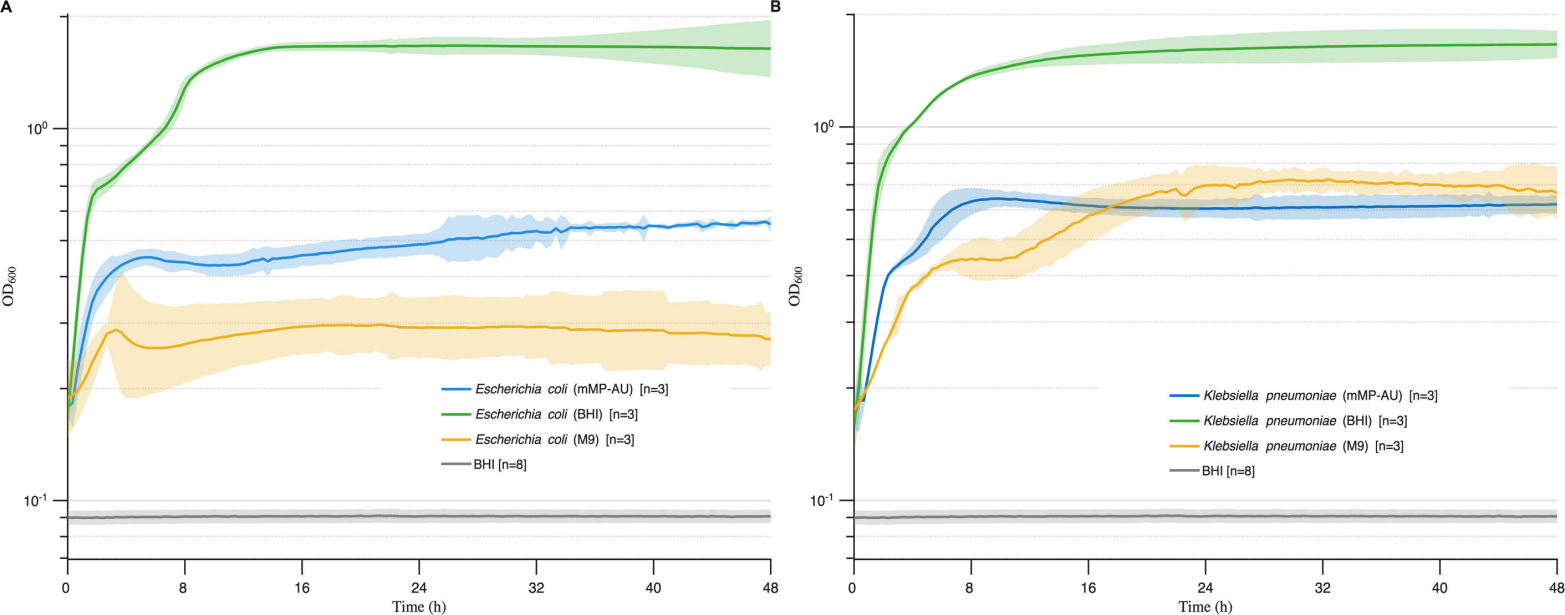
Effect of trace nutrients from complex media on bacterial growth. Overnight cultures of *E. coli* (UMB3190) (**A**) and *K. pneumoniae* (UMB9987) (**B**) were diluted 1:100 into BHI, M9, or mMP-AU and incubated for 48 hours. OD_600_ is shown on logarithmic *y*-axes, and time (hours) on *x*-axes. Solid lines represent mean values and shading standard error of the mean. OD_600_ of BHI (gray) is included for reference.

Strains of *E. coli* (UMB3190) and *K. pneumoniae* (UMB9987), pre-grown on the rich medium BHI and then transferred to mMP-AU or M9 salts without supplementation, reached between one-tenth and one-third of their maximum OD_600_ when grown in BHI, indicating that residual nutrients from the rich medium supported partial growth (Fig. 2A and B).

### SimUrine formulation optimization

#### SimUrine.v1

**Fig 3:**
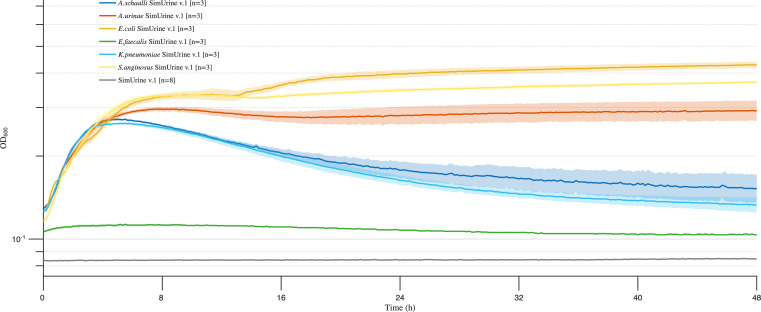
Growth in SimUrine.v1. Cultures of different bacterial isolates over 48 hours. Growth (OD_600_) is shown on a logarithmic *y*-axis, and time (hours) on the *x*-axis. Solid lines represent mean values and shading standard error of the mean. OD_600_ of SimUrine.v1 (gray) is included for reference.

This simplest version of SimUrine supported a limited growth of the facultative anaerobic bacterial species *E. coli* (UMB3190), *Actinotignum schaalii* (UMB13319), *A. urinae* (UMB5254), *K. pneumoniae* (UMB9987), and *Streptococcus anginosus* (UMB8616), while completely failing to support the growth of *E. faecalis* (Fig. 3). As further formulations allowed the growth of new organisms, this has been presented. Bacteria that grew in earlier formulations also grow in SimUrine.v6 (Table S1). Presented plots aim to lead in the rationale behind each protocol modification.

#### SimUrine.v2

**Fig 4:**
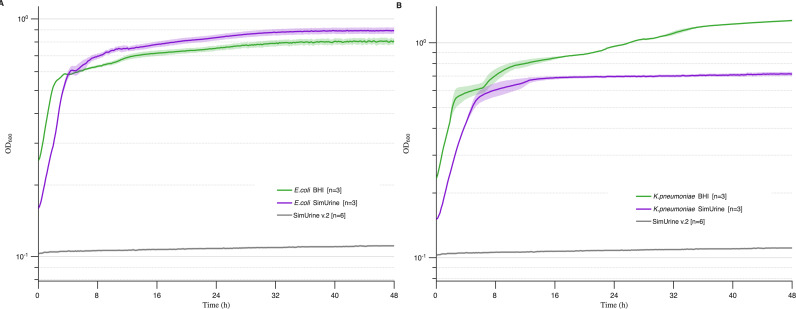
Growth in SimUrine.v2. Cultures of *E. coli* (UMB3190) (**A**) and *K. pneumoniae* (UMB9987) (**B**) in SimUrine.v2. Growth (OD_600_) is shown on logarithmic *y*-axes, and time (hours) on *x*-axes. Parallel cultures in BHI (green lines) were used as a reference. Solid lines represent mean values and shading standard error. OD_600_ of SimUrine.v2 (gray) is included for reference.

Adding vitamins and trace elements to our formulation markedly enhanced the growth of *E. coli* (UMB3190), with OD_600_ values increasing from 0.2–0.3 (Fig. 3) to 0.7, which exceeded growth in BHI (Fig. 4A). *K. pneumoniae* (UMB9987) also showed improved growth compared to SimUrine.v1 (Fig. 3 and Fig. 4B); however, unlike *E. coli*, *K. pneumoniae* did not surpass its growth in BHI. No other improvements were observed. Despite enhanced bacterial growth with this version, a precipitate formed in SimUrine.v2 after 24 hours of preparation (Fig. S3).

#### SimUrine.v3

**Fig 5:**
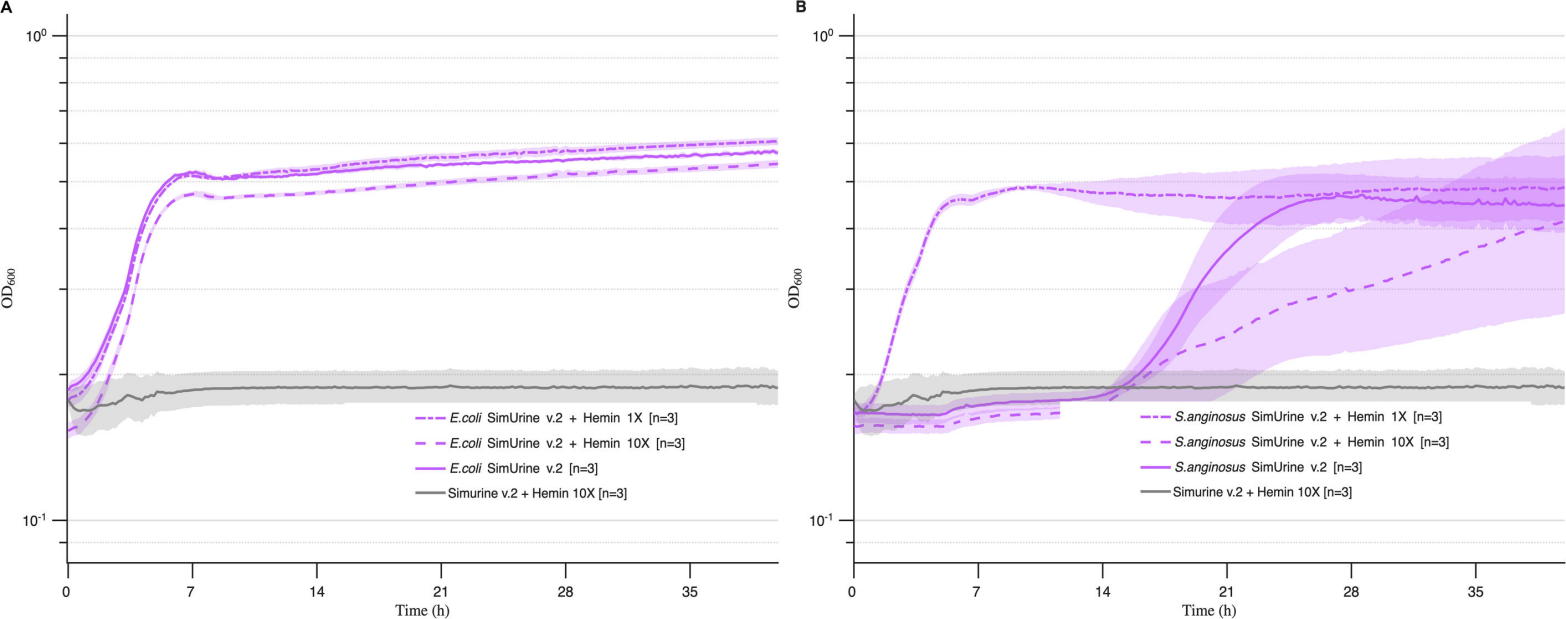
Growth in SimUrine.v2 and hemin-supplemented versions. Cultures of *E. coli* (UMB3190) (**A**) and *S. anginosus* (UMB8616) (**B**) in SimUrine.v2 (solid lines) and SimUrine.v2 supplemented with hemin 1× (dotted lines) and hemin 10× (dashed lines). Growth (OD_600_) is shown on logarithmic *y*-axes, and time (hours) on *x*-axes. Lines represent mean values and shading standard error of the mean. OD_600_ of SimUrine.v2 supplemented with 10× hemin (gray) is included for reference.

Hemin supplementation (either 1× or 10×) to produce SimUrine.v3 did not substantially improve the growth of *E. coli* (UMB3190) (compared to the growth of *E. coli* in SimUrine.v2) or allowed the growth of new species but had a marked effect on the growth characteristics of *S. anginosus* (UMB8616) (Fig. 5A and B). Based on these results, we adapted our formulation, reducing the concentration of hemin from 10× to 5×. We also evaluated the importance of uric acid for our formulation. Experiments revealed that uric acid was essential for reaching higher OD_600_ values and for enhancing exponential growth, while hemin 5× performed well (Fig. S4A). Thus, 5× hemin incorporation defined SimUrine.v3.

#### SimUrine.v4

**Fig 6:**
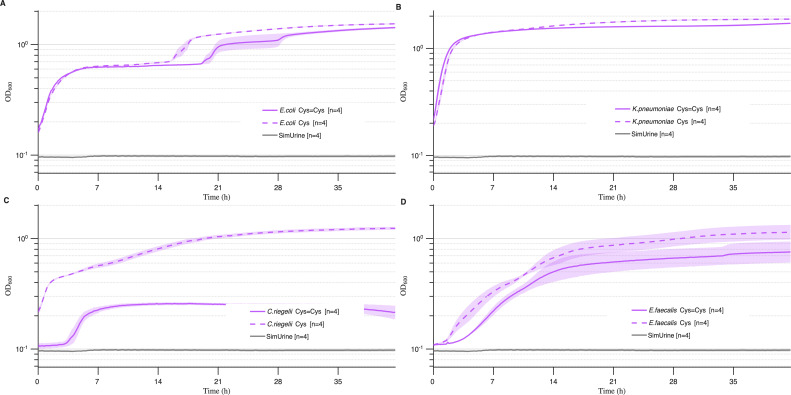
Growth in SimUrine.v4. Cultures of *E. coli* (UMB3190) (**A**), *K. pneumoniae* (UMB9987) (**B**), *C. riegelii* (UMB12267) (**C**), and *E. faecalis* (UMB3193) (**D**) in cysteine (dotted lines) or cystine (solid lines). Growth (OD_600_) is shown on logarithmic *y*-axes, and time (hours) on *x*-axes. Lines represent mean values and shading standard error of the mean. OD_600_ of SimUrine.v4 (gray) is included for reference.

SimUrine.v4 represented a major improvement of the SimUrine formulation. We incorporated amino acids within urine-relevant concentration ranges (17), changed the order of hemin and uric acid supplementation, and introduced both HEPES and Tween 80. We used this formulation to test if bacteria could grow with cysteine or its dimer, cystine. For *E. coli* (UMB3190) and *K. pneumoniae* (UMB9987), SimUrine.v4 resulted in vastly improved OD_600_ (compare Fig. 6A and B to Fig. 5A and B) and resulted in substantial growth of *E. faecalis* (UMB3193), while also allowing the growth of *Corynebacterium riegelii* (UMB12267) (Fig. 6C and D). In all cases, cysteine performed better than cystine.

#### SimUrine.v5

We increased long-term formulation stability without altering growth characteristics by replacing HEPES with MOPS, incorporating sodium bicarbonate, and by filter-sterilizing the medium (data not shown). Defining stability by the absence of precipitates, change of color, or appearance, the lifespan of the formulation was extended to a maximum of 4 weeks in a dark environment, either at room temperature (21°C) or when refrigerated (4°C).

#### SimUrine.v6

**Fig 7:**
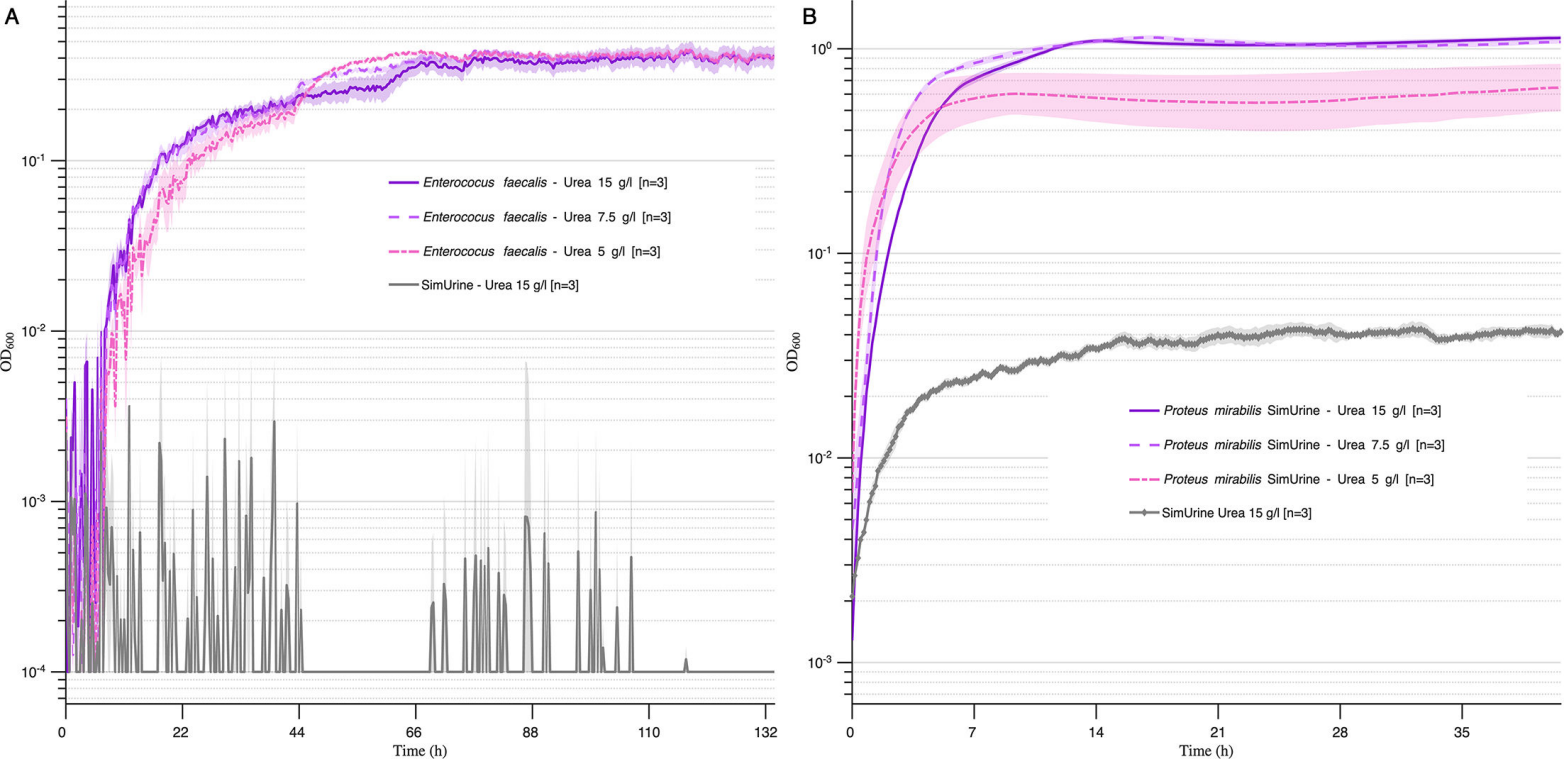
Effect of urea in SimUrine.v6. *E. faecalis* (UMB7540) (**A**) and *P. mirabilis* (strain 83) (**B**) in SimUrine.v6 with three different concentrations of urea (15 g/L, solid lines; 7.5 g/L, dashed lines; and 5 g/L, dotted lines). Growth (OD_600_) is shown on logarithmic *y*-axes, and time (hours) on *x*-axes. Lines represent mean values and shading standard error. OD_600_ of SimUrine.v6 (gray) is included for reference.

The new SimUrine.v6 formulation reduced the growth of *E. faecalis* compared to SimUrine.v4 (compare Fig. 7A to Fig. 6D). Despite this, supplementation with glucose, FeSO_4_, L-valine, L-threonine, and L-tryptophan in SimUrine.v6 supported the growth of a larger number of species than previous versions (Table S1). Urea concentration did not substantially affect the growth of *E. faecalis* (UMB3193) (Fig. 7A), but the higher urea concentrations improved the growth of *P. mirabilis* (UMB7310) (Fig. 7B). The higher urea concentration was kept in the final formulation to better reflect physiological urine levels, as bacterial viability and medium stability were not compromised. SimUrine.v6 allowed the growth of several *Lactobacillus* isolates even at pH = 5 (Table 1).

**TABLE 1:** CFU/mL and OD_600_ for *Lactobacillus* cultures in SimUrine.v6 (pH = 5)

Strain	CFU/mL (0H)	OD_600_ (0H)	CFU/mL (48H)	OD_600_ (48H)
*Lactobacillus gasseri* UMB5255	2.97 × 10^4^ (±5.77 × 10^2^)	0.081	9 × 10^5^ (±2 × 10^5^)	0.118 (±0.001)
*Lactobacillus gasseri* UMB246	3.3 × 10^3^ (±1.53 × 10^3^)	0.081	4.43 × 10^5^ (±6.4 × 10^4^)	0.095 (±0.001)
*Lactobacillus gasseri* UMB245	3 × 10^3^ (±0)	0.081	2.2 × 10^5^ (±3.4 × 10^4^)	0.103 (±0.006)
*Lactobacillus gasseri* UMB8273	bdl	0.081	3.9 × 10^5^ (±5.5 × 10^4^)	0.097 (±0.001)
*Lactobacillus gasseri* UMB8059	bdl	0.081	5.8 × 10^5^ (±5.3 × 10^4^)	0.117 (±0.009)
*Lactobacillus crispatus* UMB244	bdl	0.081	1.1 × 10^3^ (±0)	0.09 (±0.002)
*Lactobacillus crispatus* UMB576	7.3 × 10^3^ (±1.53 × 10^3^)	0.081	3.57 × 10^4^ (±2.8 × 10^3^)	0.092 (±0.002)

### Use of SimUrine.v6 in biologically relevant urobiome microbial experiments

**Fig 8:**
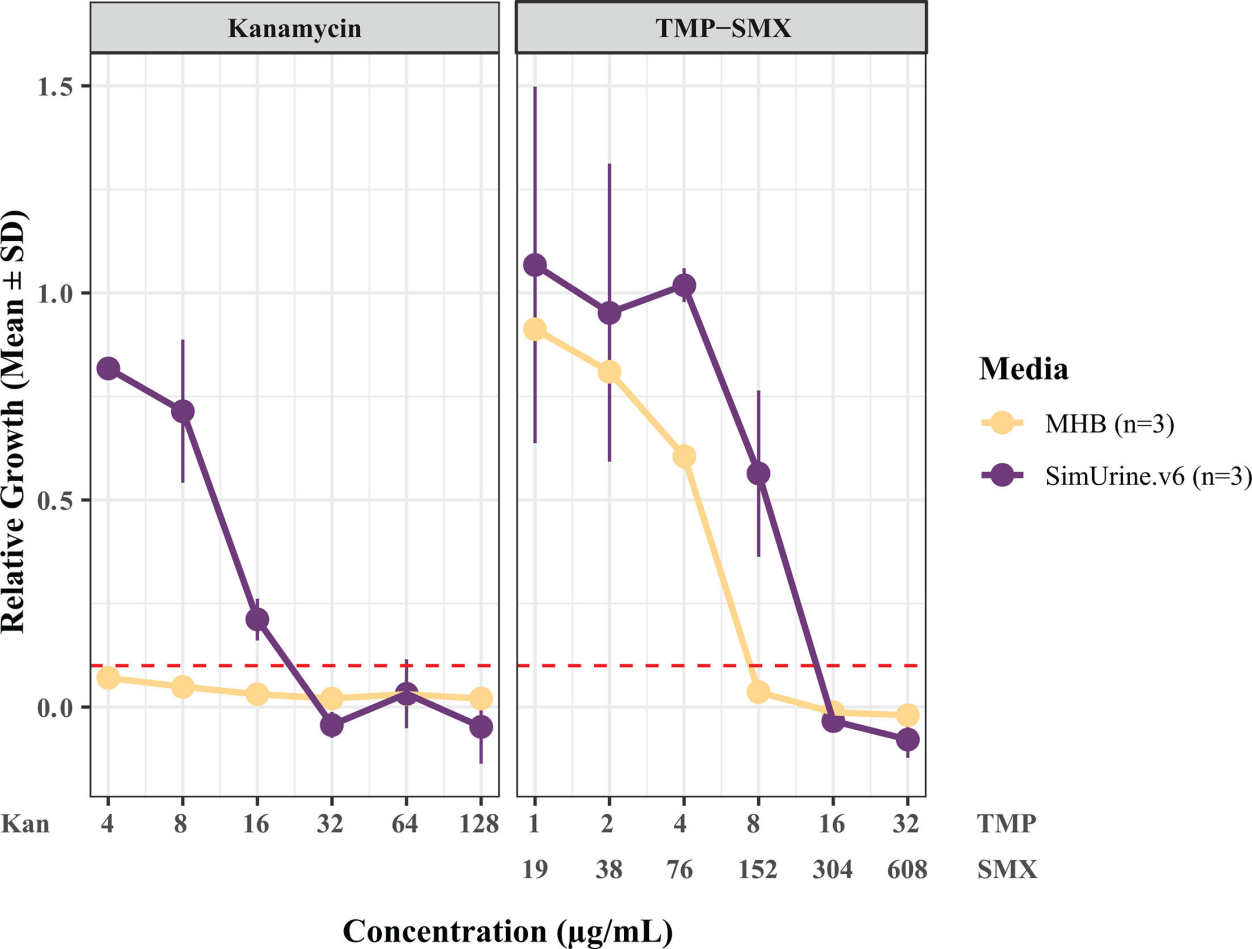
MIC determination for kanamycin (left) and TMP-SMX (right) against *E. coli* (UPEC20). Bacteria were cultured in either SimUrine or MHB medium with varying concentrations of Kan (range: 4–128 µg/mL) or TMP-SMX (range: 1/19 to 32/608 µg/mL, trimethoprim:sulfamethoxazole ratio: 1:19). Antibiotic-free cultures were used as controls. Bacterial growth was quantified by calculating the area under the curve from optical density measurements. Results were normalized to the positive control to generate the relative growth. The dashed line represents the 10% growth threshold used to define the MIC. Data points represent the mean ± SD of biological triplicates (*n* = 3).

We evaluated the use of SimUrine.v6 in two ecology-relevant contexts: antibiotic susceptibility testing and a bacterial interaction assay. *E. coli* (UPEC20) exhibited reduced susceptibility to both TMP-SMX and kanamycin in SimUrine.v6 compared to that observed in the richer, complex reference medium, MHB (Fig. 8).

**Fig 9:**
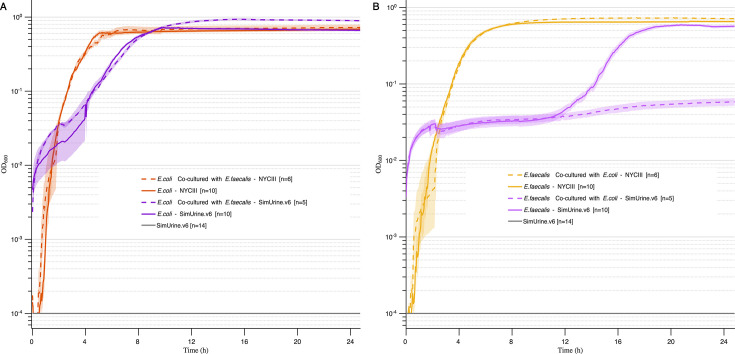
Bacterial growth curve in NYCIII medium of *E. coli* (ATCC 25922) (**A**) and *E. faecalis* (ATCC 29212) (**B**) in a dual system. Growth (OD_600_) is shown on logarithmic *y*-axes, and time (hours) on *x*-axes. Lines represent mean values and shading standard error of the mean. Solid lines represent individual cultures, and discontinuous lines represent cultures of *E. coli* (**A**) or *E. faecalis* (**B**) in opposing duet wells in SimUrine.v6. OD_600_ of SimUrine.v4 (gray) is included for reference.

Growth characteristics of *E. coli* (ATCC 25922) and *E. faecalis* (ATCC 29212) also differed in SimUrine.v6 versus the rich, complex NYCIII. In NYCIII, entry of *E. coli* into exponential growth was rapid and there was little difference in the growth characteristics of *E. coli* cultured alone and in co-culture with *E. faecalis* (Fig. 9A). In SimUrine.v6, entry of *E. coli* into exponential growth was similarly slower when grown either alone or in co-culture with *E. faecalis*, but entry of *E. coli* into stationary phase was more abrupt and occurred at a lower OD_600_ when co-cultured with *E. faecalis* (Fig. 9B). In NYCIII, entry of *E. faecalis* into exponential growth was similarly rapid when grown alone or in co-culture with *E. coli*, while entry into stationary phase occurred at a slightly lower OD_600_ when co-cultured with *E. coli* than when grown alone (Fig. 9A). In SimUrine.v6, *E. faecalis* grown alone experienced a long lag, but when co-cultured with *E. coli*, it failed to grow (Fig. 9B).

### Use of SimUrine for culturing fastidious organisms

SimUrine.v6 (pH = 5) supported the culture of multiple strains of multiple *Lactobacillus* species. Using a modified version of SimUrine.v6, we cultured strains of *L. crispatus* and *L. gasseri*. Whereas only modest changes in OD_600_ values were observed after 48H incubation, substantial changes in CFU/mL indicated growth of the evaluated strains. Some strains did not grow in CHROMagar at the initial timepoint, but they showed growth in the 48H cultures (Table 1).

### SimUrine.v6 physicochemical parameters

SimUrine.v6 exhibited physicochemical properties within the normal range of human urine (as described in the literature [Table 2]), including low viscosity, making it suitable for microfluidic applications.

**TABLE 2:** Physicochemical parameters of SimUrine.v6

Parameter (20°C)	SimUrine.v6	diH_2_O	Urine (reference)
Density (g/mL)	1.0095 (±0.0035)	0.9869	1.005–1.030 (25)
Conductivity (mS/cm)	16.9	0	1–34 (~22) (26, 27)
Refractive index	1.3404	1.333	1.34 (28)
Viscosity (mPa s)	1.1080	1.000	1.05–1.2 (29)
Osmolarity (mOsm/kg)	671.25 (±3.78)	0	500–850 (30)
Optical density (600 nm)	0.024 (±0.001)^*a*^	0	–^*b*^

^*a*^ Value after plate subtraction, contrary value = 0.081.

^*b*^ –, not applicable.

## DISCUSSION

Conventional culture media for human urobiome studies utilize standardized formulations that fail to reflect the urinary tract’s natural biochemical environment. This discrepancy stems primarily from the use of undefined reagents that create complex, proprietary formulations that are difficult to replicate across laboratories. The human bladder maintains distinct physiological parameters including specific pH, osmolarity, and metabolite concentrations that differ substantially from commercial medium compositions. We developed SimUrine, a fully chemically defined medium where each component was selected to either match the documented human biochemical parameters or support urobiome growth at minimal effective concentrations.

Following urobiome discovery, researchers have attempted to isolate, culture, and characterize these microbes, using artificial urine media or enriched formulations (e.g., NYCIII medium) to maximize isolate recovery. While these approaches have enabled the isolation and study of novel strains, current medium formulations have limited our understanding of their behavior under native conditions.

SimUrine development integrates current knowledge of urine composition, artificial urine media, and cultivation strategies used for other microbial communities, such as the gut microbiome (16). By adapting the established MP-AU medium, we successfully cultured bacteria important for bladder health and disease while providing an open, defined, and modifiable medium formulation. This medium permits the behavioral study of many fastidious and anaerobic bacteria species in conditions that simulate natural nutrient availability in urine.

Development of SimUrine followed a systematic approach, beginning with a basic salt base (MP-AU) that was subsequently enriched with multiple carbon sources to identify the most relevant biochemical requirements for bacterial growth. Our initial version (SimUrine.v1) incorporated nutrients accessible to the urobiome within the bladder environment. While N-acetylglucosamine, L-threonine, and L-serine may not be components of excreted urine, the urobiome has access to mucins and epithelial cells within the bladder lining, from where these nutrients can be metabolized (31). This formulation yielded improved growth for the tested bacteria (Fig. 3).

To enable the growth of a broader range of microbes, we selected nutrients and vitamins identified from successful gut microbiome cultivation studies. SimUrine.v2 included common vitamins and trace elements, resulting in enhanced growth of *E. coli* and *K. pneumoniae* (Fig. 4), although it did not support the optimal growth of other species of interest, such as *E. faecalis* and *A. urinae*, among others. Subsequent testing revealed that hemin incorporation and expanded amino acid availability had considerable effects on bacterial growth, as demonstrated in SimUrine.v4 (Fig. 5; Fig. S4).

The inclusion of Tween 80, an important medium surfactant, along with improved buffering capacity through HEPES addition, extended medium utility to more fastidious organisms such as *C. riegelii* (Fig. 6). However, *Lactobacillus* species remained uncultured with this formulation. Final optimization involved replacing HEPES with MOPS, adding sodium bicarbonate, implementing filter sterilization rather than autoclaving, and supplementing with FeSO_4_. We hypothesize that HEPES combined with phosphate buffer provides insufficient buffering capacity under 5% CO_2_ incubation, potentially contributing to trace element chelation that affects fastidious bacterial growth. In contrast, MOPS maintains pH stability and reduces trace element chelation, while FeSO_4_ enhances iron bioavailability.

SimUrine enables the use of physiological urea concentrations without completely suppressing the growth of urea-susceptible bacteria (Fig. 7), and urea incorporation proved beneficial for species such as *P. mirabilis* (Fig. 7B). Indeed, urea is noted to be the most abundant organic molecule in human urine at concentration ranges between 9.3 and 23.3 g/L (Putnam & McDonnell Douglas Astronautics Company-West, 1971). Thus, at 15 g/L, urea concentration in SimUrine allows for a more accurate representation of a natural bladder environment, while maintaining broad bacterial compatibility.

With our final formulation, we could culture *Lactobacillus* species in glass and plastic tubes (Table S1); however, we were not able to obtain reliable growth curves using 96-well plates, only discrete changes in OD_600_ values. To obtain these growth curves, we reduced the pH of SimUrine.v6 from 6.0 to 5.0, and we repeated the experiments, also counting CFU/mL. With this approach, the OD_600_ values did not increase substantially, but the CFU/mL indicated actual growth of *Lactobacillus* species (Table 1). These results highlight the relevance of SimUrine for the study of the urobiome, as SimUrine allows the culturing of fastidious bacteria at very low optical density, which aligns with expected growth for these organisms in the natural niche. The beneficial use of SimUrine in research does not relate to abundant growth yields, but rather in mimicking the natural environment. Thus, reliance only on OD_600_ values may not be appropriate when testing the growth of slow-growing and/or fastidious organisms in our urine-like medium.

Beyond the logical importance of using a medium that better replicates actual urine composition, our antibiotic susceptibility and co-culture results demonstrate the feasibility of utilizing SimUrine in clinically oriented research. Using this fully defined medium, for the tested strain (Fig. 8), we detected lower antibiotic susceptibility than standard values for *E. coli* (32), even when using *in vivo*-like TMP-SMX proportions (33), potentially reflecting susceptibilities closer to those encountered under native bladder conditions, but this requires further investigation. Notwithstanding these observations, the complexity of our medium may present a barrier to its use for clinical diagnosis, but it certainly can be used to evaluate the impact of therapies in a more defined context that better mimics the bladder environment. For example, SimUrine enabled observation of previously unknown interactions between urobiome community members, highlighting the importance of physiologically relevant culture conditions for understanding clinical microbiology.

SimUrine represents a fully defined culture medium that supports the growth of diverse bacterial species and clinical isolates while remaining adaptable to specific research and clinical requirements. The medium’s complete chemical definition ensures reproducibility across laboratories, while its modular design allows targeted modifications without compromising stability. Previous artificial urine compositions, such as those formulated by Brooks and Keevil or Zandbergen, utilize protein hydrolysates that provide wide coverage of necessary amino acids but introduce unnecessary variability via undefined compositions (11, 12). With defined nutrient compositions, the dietary behaviors of tested bacteria can be fully characterized with SimUrine. We acknowledge the complexity of our formulation, but that is the nature of urine. Our intent has been to provide a formulation whose composition can be modified. Thus, we included the most relevant results for each step of its formulation, such that researchers can implement possible modifications based on their needs.

The clinical implications of this work extend beyond improved pathogen recovery and characterization. By providing culture conditions that more accurately reflect the native bladder environment, SimUrine enables more clinically relevant assessment of antimicrobial susceptibility patterns (Fig. 8) and microbial interactions that may not be observed when using rich media (Fig. 9) (32). We acknowledge that the *E. faecalis* suppression in co-culture needs mechanistic investigation, which exceeds the purpose of this work.

This enhanced physiological relevance may lead to improved therapeutic decision-making and better understanding of urobiome dynamics in health and disease. The defined nature of SimUrine medium, and its physicochemical properties (Table 2), makes it ideal for use in the study of the urobiome, using the duet system, microfluidic devices, organoid-on-a-chip devices, or simple flasks; its use will depend on the nature of the research questions (Fig. S5).

SimUrine cannot fully simulate several aspects of human urine. Certainly, it would be nearly impossible to account for the thousands of biomolecules that can be isolated in human urine, the majority of which are found in near-trace amounts. However, even the most minute quantities of certain compounds may have physiological relevance when studying urobiome behaviors, SimUrine, by its fully defined nature, enables the assessment of such physiologically relevant molecules or compounds. SimUrine excludes hormones, xenobiotics, chromogens, cellular debris, mucoproteins, and macromolecules (DNA, collagen) due to instability, undefined composition, wide physiological ranges, or cost. However, SimUrine attempts to account for the vital contribution of glycosaminoglycans in the bladder environment toward bacterial survival with the inclusion of N-acetylglucosamine, L-threonine, and L-serine, all glycosaminoglycan degradation metabolites. It will be important to account for these missing variables when attempting to simulate bladder environments. Incorporating cell cultures such as urothelial cell lines or organoids may attempt to address some of these deficits.

Future applications of SimUrine include standardized urobiome research protocols, probiotic efficacy testing under physiologically relevant conditions, and development of personalized therapeutic approaches based on individual urine chemistry profiles. The medium’s defined composition also facilitates mechanistic studies of host-microbe interactions. We expect that SimUrine will be beneficial to anyone doing urobiome research and will serve as a tool for better understanding bacterial interactions within the urinary tract ecosystem.

## SUPPLEMENTAL MATERIAL

Protocol S1 aem.01559-25-s0001.docx SimUrine.v6 protocol.

Protocol S2 aem.01559-25-s0002.docx SimUrine.v1 to SimUrine.v5 protocols.

Supplemental Material aem.01559-25-s0003.docx Figures S1 to S5; Tables S1 and S2.

ASM does not own the copyrights to Supplemental Material that may be linked to, or accessed through, an article. The authors have granted ASM a non-exclusive, world-wide license to publish the Supplemental Material files. Please contact the corresponding author directly for reuse.
