# Supplementary material for: SimUrine: a novel, fully defined artificial urinary medium for enhanced microbiological research of urinary bacteria
Source: Appl Environ Microbiol. 2026 Jan 9;92(2):e01559-25. doi: 10.1128/aem.01559-25 (PMC12915295; doi:10.1128/aem.01559-25)
Supplement: Protocol S1 — SimUrine.v6 protocol. [file aem.01559-25-s0001.docx]

**Supplementary Protocols 1**

**SimUrine.v6**

Protocol for 500mL* of SimUrine.v6 1X.

Add 200 mL of H_2_O to a flask and dissolve the following reagents one by one stirring and keeping at 40°C.

| **Module** | **Reagent** | **g** | **g/L** | **Concentration (mM)** |
| --- | --- | --- | --- | --- |
| Core salts  (mMP-AU) | Na_2_SO_4_ | 0.85 | 1.7 | 11.97 |
|  | NaCl | 0.878 | 1.756 | 30.05 |
|  | Na_2_HPO_4_ | 0.3315 | 0.663 | 4.67 |
|  | NH_4_Cl | 0.75 | 1.5 | 28.04 |
|  | NaH_2_PO_4_*H_2_O | 1.29 | 2.58 | 18.67 |
|  | KCl | 1.154 | 2.308 | 30.96 |
|  | Creatinine | 0.44 | 0.88 | 7.78 |
|  | NH_4_SO_4_*7H_2_O | 0.014 | 0.028 | 0.114 |
|  | Potassium citrate monohydrated | 0.513 | 1.026 | 3.16 |
| Amino Acids | L-cysteine | 0.25 | 0.5 | 4.17 |
|  | L-valine | 0.25 | 0.5 | 4.27 |
|  | L-tryptophan | 0.25 | 0.5 | 2.45 |
|  | L-threonine | 0.75 | 1.5 | 12.59 |
| Carbon Sources | n-acetyl-glucosamine | 2.765 | 5.53 | 25 |
|  | D-glucose | 0.1 | 0.2 | 1.11 |
|  | Lactic acid | 150 µL | 300 µL | 4.02 |
|  | Pyruvic acid | 8.92 µL | 17.84 µL | 0.253 |
|  | Acetic acid | 8.92 µL | 17.84 µL | 0.312 |
| Others | FeSO_4_ (1mM) | 500 µL | 1 mL | 0.001 |
|  | Ascorbic acid | 0.05 | 0.1 | 0.568 |
|  | NaHCO_3_ | 0.084 | 0.168 | 2 |

While media is hot, add 500 µL of Tween-80.

Add 1.05g of MOPS

Add 7.5 g of Urea

*Minimal recommended volume to prevent compositional variability.

Add:

1. 0.5 mL of solution of trace elements 1 (Acid)
2. 0.5 mL of solution of trace elements 2 (Basic)
3. 5 mL of vitamin mix 1
4. 5 mL of vitamin mix 2
5. 2 mL of solution of MgSO_4_ 108g/L (0.216 g)_._
6. 1 mL solution of NH_4_C_2_O_4_ 13.5 g/L (0.0135 g).
7. 100 µL of solution of **MnSO_4_** 0.8 g/L.
8. 5 mL of amino acids 100X solution.
9. 2500 µL of hemin solution 0.5 g/L (0.00125 g).

**Check pH (it should be ~ pH= 6-7), since uric acid was dissolved in NaOH 10M, adding next solution will reduce pH and then I can be adjusted.**

1. 10 mL of uric acid solution 12.5 g/L (0.125 g).

**Check pH (it should be ~ pH= 6, adapt it to pH=6 with HCl)**

1. Add 0.25 g of L-serine

**Complete volume to 500 mL with H_2_O. Filter using 0.2 µm filter. pH should stay stable.**

**SOLUTIONS**

The following solutions must be prepared and sterilized before using them.

1. **Trace elements (Acid). (Filter using a 0.2 µm filter. Protect from light, 4°C or RT)**

| Reagent | mg | concentration |
| --- | --- | --- |
| CuCl_2_ * 2H_2_O | 1.70 | 0.1 mM |
| H_3_BO_3_ | 6.18 | 1 mM |
| CoCl_2_*6H_2_O | 11.89 | 0.5 mM |
| NiCl_2_*6H_2_O | 2.38 | 0.1 mM |
| ZnCl_2_ | 6.82 | 0.5 mM |
| FeCl_2_*4H_2_O | 149.11 | 7.5 mM |

Dissolve in 100 mL of water and add 8.3 µL of HCl 37%.

1. **Trace elements (Basic) (Filter using a 0.2 µm filter. 4°C or RT)**

| Reagent | mg | concentration |
| --- | --- | --- |
| Na_2_SeO_3_ | 1.73 | 0.1 mM |
| Na_2_WO_4_*2H_2_O | 3.3 | 0.1 mM |
| Na_2_MoO_4_*2H_2_O | 2.42 | 0.1 mM |

Dissolve in 100 mL of NaOH 10 mM

1. **Vitamins 1 (Filter using a 0.2 µm filter. Protect from light, 4°C)**

| Reagent | mg |
| --- | --- |
| Biotin | 2 |
| Nicotinamide | 20 |
| Thiamine | 20 |
| Riboflavin | 10 |

Dissolve in 100 mL of solution CaCl_2_ (15.25 g/L)

1. **Vitamins 2 (Filter using a 0.2 µm filter. Protect from light, 4°C)**

| Reagent | mg |
| --- | --- |
| p-aminobenzoic acid | 10 |
| Pantothenic acid | 10 |
| pyridoxamine | 50 |
| Cyanocobalamin | 10 |
| Folic acid | 10 |

Dissolve in 100 mL of solution CaCl_2_ (15.25 g/L)

1. **MgSO_4_ solution (Autoclave or filter using a 0.2 µm filter. RT or 4°C. Indef. Storage)**

MgSO₄·7H₂O 5.4 g in 50 mL of H_2_O

1. **NH_4_C_2_O_4_ solution (Autoclave or filter. Indef. Storage)**

NH_4_C_2_O_4_·H_2_O 1.35 g in 100 mL of H_2_O

1. **MnSO4 solution (Indef. Storage)**

MnSO_4_ 0.08 g in 100 mL of H_2_O.

1. **Amino acids 100X solution (100 mL)**

Add 40 mL of H_2_O and incorporate the water-soluble amino acids one by one. Dissolve individually the other amino acids in ~ 5mL of HCl 1M and add to the bottle. Complete volume to 100 mL with H_2_O. To ensure solubility, **keep solution at pH ~1**.

| **Amino acid** | **Concentration mM** | **mg** | **Solubility** |
| --- | --- | --- | --- |
| Arginine | 0.13 | 226.40 | H_2_O |
| Histidine | 0.86 | 884.40 | H_2_O |
| Isoleucine | 0.075 | 98.30 | **HCl** |
| Alanine | 0.55 | 489.90 | H_2_O |
| Asparagine | 0.094 | 124.20 | H_2_O |
| Aspartate | 0.63 | 838.53 | H_2_O |
| Cystine | 0.35 | 841.05 | **HCl** |
| Glutamate | 1.46 | 2148.00 | H_2_O |
| Glutamine* | 0.53 | 774.50 | H_2_O |
| Glycine | 4.27 | 3205.40 | H_2_O |
| Leucine | 0.113 | 148.20 | **HCl** |
| Lysine | 0.27 | 394.70 | **HCl** |
| Methionine | 0.046 | 68.63 | **HCl** |
| Phenylalanine | 0.09 | 148.67 | **HCl** |
| Proline | 0.26 | 299.30 | H_2_O |
| Serine | 0.28 | 294.20 | H_2_O |

*****Lifespan of Glutamine may be reduced over time, preparation of frozen aliquots of this amino acids 100X solution may be optimal **(optional).**

1. **Hemin solution (Autoclave, protect from light, 4°C).**

Add 50 mg of Hemin in 1 mL of NaOH 1N, complete volume to 100 mL with H_2_O.

1. **Uric acid solution (Autoclave, 4 days at 4°C)**

0.625 g in 5 mL NaOH 10 M, dissolve, and complete to 50 mL with H_2_O. (Uric acid will fully dissolve after autoclaving the solution).

**Supplementary Table 2: List of reagents utilized in this work.**

| **Correlative** | **Reagents** | **Code** | **Company** |
| --- | --- | --- | --- |
| 1 | Acetic acid | A6283-1L | Sigma-Aldrich |
| 2 | Alanine | 11442468 | Thermo scientific Chemicals |
| 3 | Arginine | A5006-100G | Sigma-Aldrich |
| 4 | Asparagine | A0884-25G | Sigma-Aldrich |
| 5 | Aspartate | A9256-100G | Sigma-Aldrich |
| 6 | Biotin | B4501-100MG | Sigma-Aldrich |
| 7 | CaCl_2_ | C1016-100G | Sigma-Aldrich |
| 8 | Citric acid | C0759-100G | Sigma-Aldrich |
| 9 | CoCl_2_*6H_2_O | 202185-25G | Sigma-Aldrich |
| 10 | Creatinine | 228940500 | Thermo scientific Chemicals |
| 11 | CuCl_2_ * 2H_2_O | 10588502 | Thermo scientific Chemicals |
| 12 | Cyanocobalamin | 1.2459201 | Sigma-Aldrich |
| 13 | Cystine | 11414814 | Thermo scientific Chemicals |
| 14 | FeCl_2_*4H_2_O | 10348150 | Thermo scientific Chemicals |
| 15 | Folic acid | F7876-10G | Sigma-Aldrich |
| 16 | Glutamate | 10502751 | Thermo scientific Chemicals |
| 17 | Glutamine | 11482277 | Thermo scientific Chemicals |
| 18 | Glycine | 1.00590.1000 | MERK |
| 19 | H_3_BO_3_ | B0394-100G | Sigma-Aldrich |
| 20 | Hemin | 10499291 | Thermo scientific Chemicals |
| 21 | HEPES 1M | H3375-25G | Sigma-Aldrich |
| 22 | Histidine | H8000-5G | Sigma-Aldrich |
| 23 | Isoleucine | I2752-1G | Sigma-Aldrich |
| 24 | KCl | 1.04936.1000 | MERK |
| 25 | L-cysteine | A10435.18 | Thermo scientific Chemicals |
| 26 | L-serine | S4500-1G | Sigma-Aldrich |
| 27 | L-threonine | 1.0841101 | Sigma-Aldrich |
| 28 | Lactic acid | 10665302 | Thermo scientific Chemicals |
| 29 | Leucine | L8000-25G | Sigma-Aldrich |
| 30 | Lysine | L5501-10MG | Sigma-Aldrich |
| 31 | Methionine | M9625-25G | Sigma-Aldrich |
| 32 | MgSO_4_*7H_2_O | 447155000 | Thermo scientific Chemicals |
| 33 | MnSO_4_*H_2_O | 11478237 | Thermo scientific Chemicals |
| 34 | n-acetyl-glucosamine | 11438770 | Thermo scientific Chemicals |
| 35 | Na_2_HPO_4_ | 1.06586.0500 | Sigma-Aldrich |
| 36 | Na_2_MoO_4_*2H_2_O | 10164830 | Thermo scientific Chemicals |
| 37 | Na_2_SO_4_ | 1.28211.1001 | Sigma-Aldrich |
| 38 | Na_2_WO_4_*2H_2_O | 10117423 | Thermo scientific Chemicals |
| 39 | NaCl | 207790010 | ACROS |
| 40 | NaH_2_PO_4_ | 1.06349.1000 | Sigma-Aldrich |
| 41 | NaOH | S5881-1K | Sigma-Aldrich |
| 42 | Na_2_SeO3 | 214485-5G | Sigma-Aldrich |
| 43 | NH_4_C_2_O_4_ | 10054013 | Thermo scientific Chemicals |
| 44 | NH_4_Cl | A4514-500G | Sigma-Aldrich |
| 45 | NH_4_SO_4_*7H_2_O | A5132-1K | Sigma-Aldrich |
| 46 | NiCl_2_*6H_2_O | 654507-5G | Sigma-Aldrich |
| 47 | Nicotinamide | 10447710 | Thermo scientific Chemicals |
| 48 | p-aminobenzoic acid | A9878-5G | Sigma-Aldrich |
| 49 | Pantothenic acid | 21210-5G-F | Sigma-Aldrich |
| 50 | Phenylalanine | 11468840 | Thermo scientific Chemicals |
| 51 | Proline | 11439512 | Thermo scientific Chemicals |
| 52 | pyridoxamine | 10785052 | Thermo scientific Chemicals |
| 53 | Pyruvic acid | 10562721 | Thermo scientific Chemicals |
| 54 | Riboflavin | 10593871 | Thermo scientific Chemicals |
| 55 | Serine | 11489113 | Thermo scientific Chemicals |
| 56 | Thiamine | 10477540 | Thermo scientific Chemicals |
| 57 | Tween80 | P4780-500ML | Sigma-Aldrich |
| 58 | Urea | U5378-1K | Sigma-Aldrich |
| 59 | Uric acid | U2625-100G | Sigma-Aldrich |
| 60 | ZnCl_2_ | 208086-5G | Sigma-Aldrich |
