# Supplementary material for: SimUrine: a novel, fully defined artificial urinary medium for enhanced microbiological research of urinary bacteria
Source: Appl Environ Microbiol. 2026 Jan 9;92(2):e01559-25. doi: 10.1128/aem.01559-25 (PMC12915295; doi:10.1128/aem.01559-25)
Supplement: Protocol S2 — SimUrine.v1 to SimUrine.v5 protocols. [file aem.01559-25-s0002.docx]

**Supplementary Protocols 2**

**SimUrine.v1**

**Protocol for 500 ml of media 1X.**

Add 200 ml of H_2_O to a bottle and add the following reagents one by one.

| **Reagent** | **g** | **g/L** |
| --- | --- | --- |
| Na_2_SO_4_ | 0.85 | 1.7 |
| NaCl | 0.878 | 1.756 |
| Na_2_HPO_4_ | 0.3315 | 0.663 |
| NH_4_Cl | 0.633 | 1.266 |
| NaH_2_PO_4_ | 1.12 | 2.24 |
| KCl | 1.154 | 2.308 |
| n-acetyl-glucosamine | 2.765 | 5.53 |
| L-threonine  CaCl_2_•H2O | 0.75  0.122 | 1.5  0.244 |

Dissolve individually in 5 ml of HCl 1M and add to the media.

0.25 g L-cysteine

0.25 g L-serine

Add: Lactic acid 75 µL, Pyruvic acid 4.46 µL, Acetic acid 4.46 µL

Dissolve individually in 20 ml of H_2_O and add to the media:

Creatinine 0.44 g, MgSO_4_*7H2O 0.54 g, NH_4_SO_4_*7H2O 0.014 g,

Potassium citrate 0.484 g

Add 60 ml of H_2_O to complete ~350 ml.

Autoclave (adjust pH 6.5)

Add 10 ml of uric acid solution and 1 ml solution of NH_4_C_2_O_4_ (See Supplementary protocol 1).

Add 7.5 g of urea, adjust pH to 6.5, complete with sterile water, and filter.

**SimUrine.v2**

**Protocol for 500 ml of media 1X.**

Add 200 ml of H_2_O to a bottle and add the following reagents one by one.

| **Reagent** | **g** | **g/L** |
| --- | --- | --- |
| Na_2_SO_4_ | 0.85 | 1.7 |
| NaCl | 0.878 | 1.756 |
| Na_2_HPO_4_ | 0.3315 | 0.663 |
| NH_4_Cl | 0.633 | 1.266 |
| NaH_2_PO_4_ | 1.12 | 2.24 |
| KCl | 1.154 | 2.308 |
| n-acetyl-glucosamine | 2.765 | 5.53 |
| L-threonine | 0.75 | 1.5 |

Dissolve individually in 5 ml of HCl 1M and add to the media.

0.25 g L-cysteine

0.25 g L-serine

Add: Lactic acid 75 µL, Pyruvic acid 4.46 µL, Acetic acid 4.46 µL

Dissolve individually in 20 ml of H_2_O and add to the media:

Creatinine 0.44 g, MgSO_4_*7H2O 0.54 g, NH_4_SO_4_*7H2O 0.014 g,

Potassium citrate 0.484 g

Add 60 ml of H_2_O to complete ~350 ml.

Autoclave (adjust pH 6.5)

Add 5 ml of vitamin mix 1, 5 ml of vitamin mix 2*, 1 ml solution of NH_4_C_2_O_4_, 0.5 ml of solution of trace elements 1 (Acid), 0.5 ml of solution of trace elements 2 (Basic). (See **Supplementary Protocol 1**).

Add 10 ml of uric acid solution (12.5 g/L).

Add 7.5 g of urea, adjust pH to 6.5, complete with sterile water, and filter.

**SimUrine.v3**

**Protocol for 500 ml of media 1X.**

Add 200 ml of H_2_O to a bottle and add the following reagents one by one.

| **Reagent** | **g** | **g/L** |
| --- | --- | --- |
| Na_2_SO_4_ | 0.85 | 1.7 |
| NaCl | 0.878 | 1.756 |
| Na_2_HPO_4_ | 0.3315 | 0.663 |
| NH_4_Cl | 0.633 | 1.266 |
| NaH_2_PO_4_ | 1.12 | 2.24 |
| KCl | 1.154 | 2.308 |
| n-acetyl-glucosamine | 2.765 | 5.53 |
| L-threonine | 0.75 | 1.5 |

Dissolve individually in 5 ml of HCl 1M and add to the media.

0.25 g L-cysteine

0.25 g L-serine

Add: Lactic acid 75 µL, Pyruvic acid 4.46 µL, Acetic acid 4.46 µL

Dissolve individually in 20 ml of H_2_O and add to the media:

Creatinine 0.44 g, MgSO_4_*7H2O 0.54 g, NH_4_SO_4_*7H2O 0.014 g

Potassium citrate 0.484 g

Add 60 ml of H_2_O to complete ~350 ml.

Autoclave (adjust pH 6.5)

Add 5 ml of vitamin mix 1, 5 ml of vitamin mix 2*, 1 ml solution of NH_4_C_2_O_4_, 0.5 ml of solution of trace elements 1 (Acid), 0.5 ml of solution of trace elements 2 (Basic), 2500 µL of hemin solution (See Supplementary protocol 1).

Add 10 ml of uric acid solution (12.5 g/L).

Add 7.5 g of urea, adjust pH to 6.5, complete with sterile water, and filter.

**SimUrine.v4**

**Protocol for 500 ml of media 1X.**

Add 200 ml of H_2_O to a bottle and add the following reagents one by one.

| **Reagent** | **g** | **g/L** |
| --- | --- | --- |
| Na_2_SO_4_ | 0.85 | 1.7 |
| NaCl | 0.878 | 1.756 |
| Na_2_HPO_4_ | 0.3315 | 0.663 |
| NH_4_Cl | 0.633 | 1.266 |
| NaH_2_PO_4_ | 1.12 | 2.24 |
| KCl | 1.154 | 2.308 |
| n-acetyl-glucosamine | 2.765 | 5.53 |
| L-threonine  L-cysteine | 0.75  0.25 | 1.5  0.5 |

Dissolve individually in 5 ml of HCl 1M and add to the media.

0.25 g L-cysteine

0.25 g L-serine

Add: Lactic acid 75 µL, Pyruvic acid 4.46 µL, Acetic acid 4.46 µL

Dissolve individually in 20 ml of H_2_O and add to the media:

Creatinine 0.44 g, NH_4_SO_4_*7H2O 0.014 g

Potassium citrate 0.484 g

Add 60 ml of H_2_O to complete ~350 ml.

Autoclave (adjust pH 6.5)

**After autoclaving, add:**

(Here and while media is hot, add Tween-80 1 ml per liter for *Lactobacillus* growth.)

1. 0.5 ml of solution of trace elements 1 (Acid)
2. 0.5 ml of solution of trace elements 2 (Basic)
3. 5 ml of vitamin mix 1
4. 5 ml of vitamin mix 2
5. 1 ml of solution of MgSO_4_
6. 1 ml solution of NH_4_C_2_O_4_.
7. 100 µL of solution of MnSO_4_.

Adjust pH to 6.5 (~100µL of NaOH 10M)

**(Check/adjust pH (6.5) and add 5 ml of HEPES 1M)**

Add 7.5 g of **urea** and filter.

**After filtering**, add:

1. 5 ml of amino acids 10X solution.
2. 2500 µL of hemin solution. (check pH, add ~500 µL HCl 1M. Final pH 6.5)
3. 10 ml of uric acid solution.

**Complete volume to 500 ml with autoclaved H_2_O. pH should stay stable.**

**SimUrine.v5.**

**Protocol for 500 ml of media 1X.**

Add 200 ml of H_2_O to a bottle and add the following reagents one by one.

| **Reagent** | **g** | **g/L** |
| --- | --- | --- |
| Na_2_SO_4_ | 0.85 | 1.7 |
| NaCl | 0.878 | 1.756 |
| Na_2_HPO_4_ | 0.3315 | 0.663 |
| NH_4_Cl | 0.633 | 1.266 |
| NaH_2_PO_4_ | 1.12 | 2.24 |
| KCl | 1.154 | 2.308 |
| n-acetyl-glucosamine | 2.765 | 5.53 |
| L-threonine  L-cysteine | 0.75  0.25 | 1.5  0.5 |

Dissolve individually in 5 ml of HCl 1M and add to the media.

0.25 g L-cysteine

0.25 g L-serine

Add: Lactic acid 75 µL, Pyruvic acid 4.46 µL, Acetic acid 4.46 µL

Dissolve individually in 20 ml of H_2_O and add to the media:

Creatinine 0.44 g, NH_4_SO_4_*7H2O 0.014 g

Potassium citrate 0.484 g

Add 60 ml of H_2_O to complete ~350 ml.

Autoclave (adjust pH 6.5)

**After autoclaving, add:**

(Here and while media is hot, add Tween-80 1 ml per liter for *Lactobacillus* growth.)

1. 0.5 ml of solution of trace elements 1 (Acid)
2. 0.5 ml of solution of trace elements 2 (Basic)
3. 5 ml of vitamin mix 1
4. 5 ml of vitamin mix 2
5. 1 ml of solution of MgSO_4_
6. 1 ml solution of NH_4_C_2_O_4_.
7. 100 µL of solution of MnSO_4_.

Adjust pH to 6 (~100µL of NaOH 10M)

**(Check/adjust pH (6) and add MOPS 2.1 g/L)**

Add **2.5 g** of **urea** and filter.

**After filtering**, add:

1. 5 ml of amino acids 100X solution.
2. 2500 µL of hemin solution. (check pH, add ~500 µL HCl 1M. Final pH 6.5)
3. 10 ml of uric acid solution.

**Complete volume to 500 ml with autoclaved H_2_O. pH should stay stable.**
