# Supplementary material for: SimUrine: a novel, fully defined artificial urinary medium for enhanced microbiological research of urinary bacteria
Source: Appl Environ Microbiol. 2026 Jan 9;92(2):e01559-25. doi: 10.1128/aem.01559-25 (PMC12915295; doi:10.1128/aem.01559-25)
Supplement: Supplemental Material — Figures S1 to S5; Tables S1 and S2. [file aem.01559-25-s0003.docx]

**Supplementary Data**


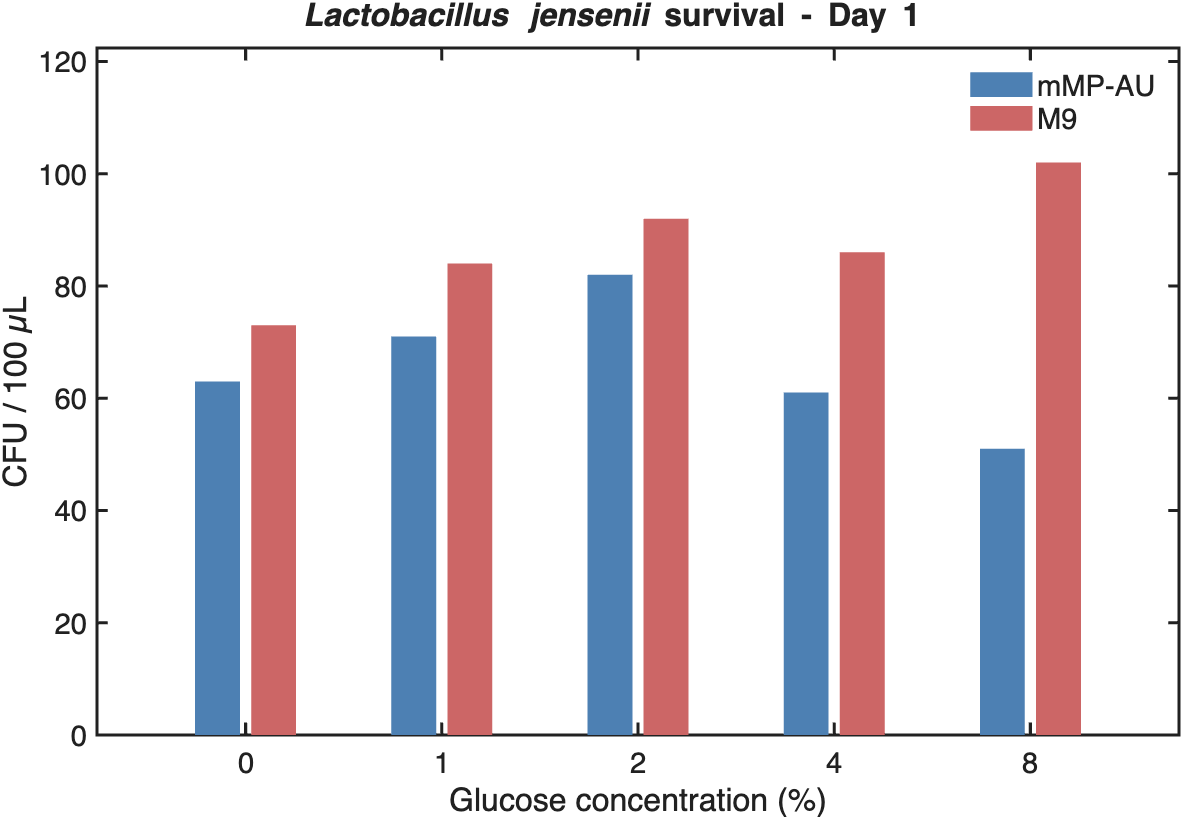

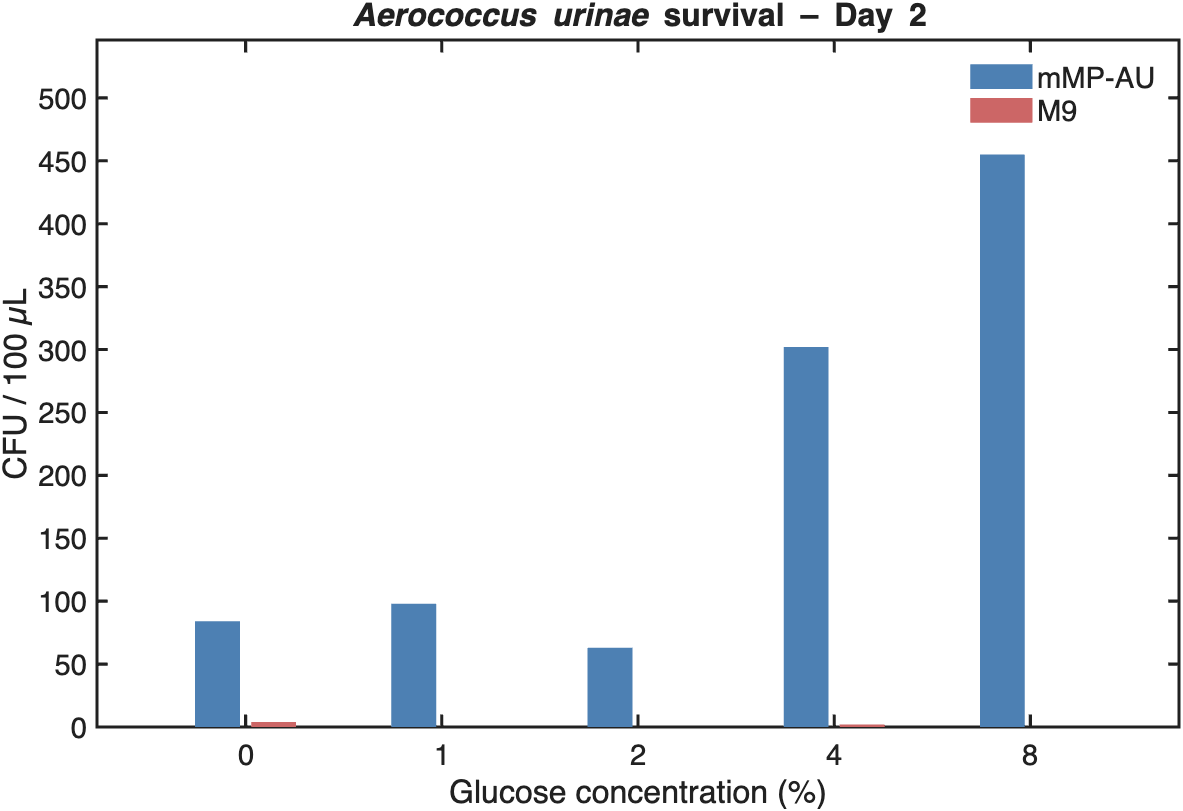


**Supplementary Figure 1 | Effect of glucose supplementation on bacterial survival.** (**A**) Lactobacillus jensenii (UMB8651) and (**B**) Aerococcus urinae (UMB5254) survival in mMP-AU or M9 salts supplemented with glucose. The y-axes display CFU/100 µL, and the x-axes represent glucose concentration.


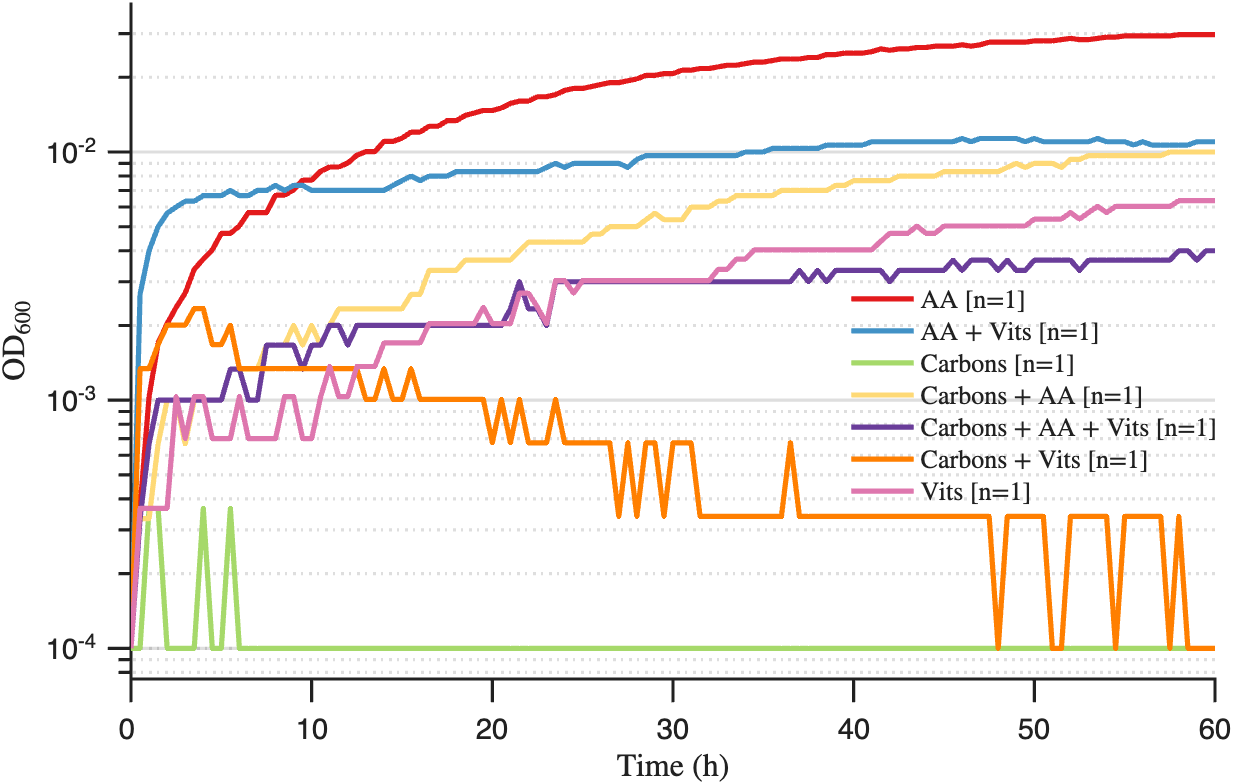


**Supplementary Figure 2 | Effect of nutrient supplementation on L. jensenii growth.** Growth curves of L. jensenii (UMB8651) cultured in mMP-AU supplemented with carbon sources, amino acid sources, vitamins, or their combinations. Optical density at 600 nm (OD_600_) is shown on the logarithmic y-axis; time (hours) on the x-axis.


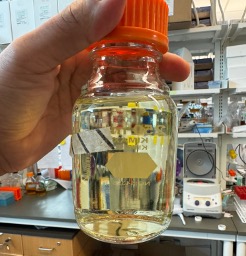

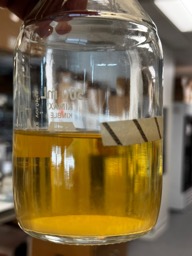


**Supplementary Figure 3**: **SimUrine.v1**. Left image shows the medium as it looked just out of the autoclave; right image, after 24 hours. Precipitate was formed within 24 hours.


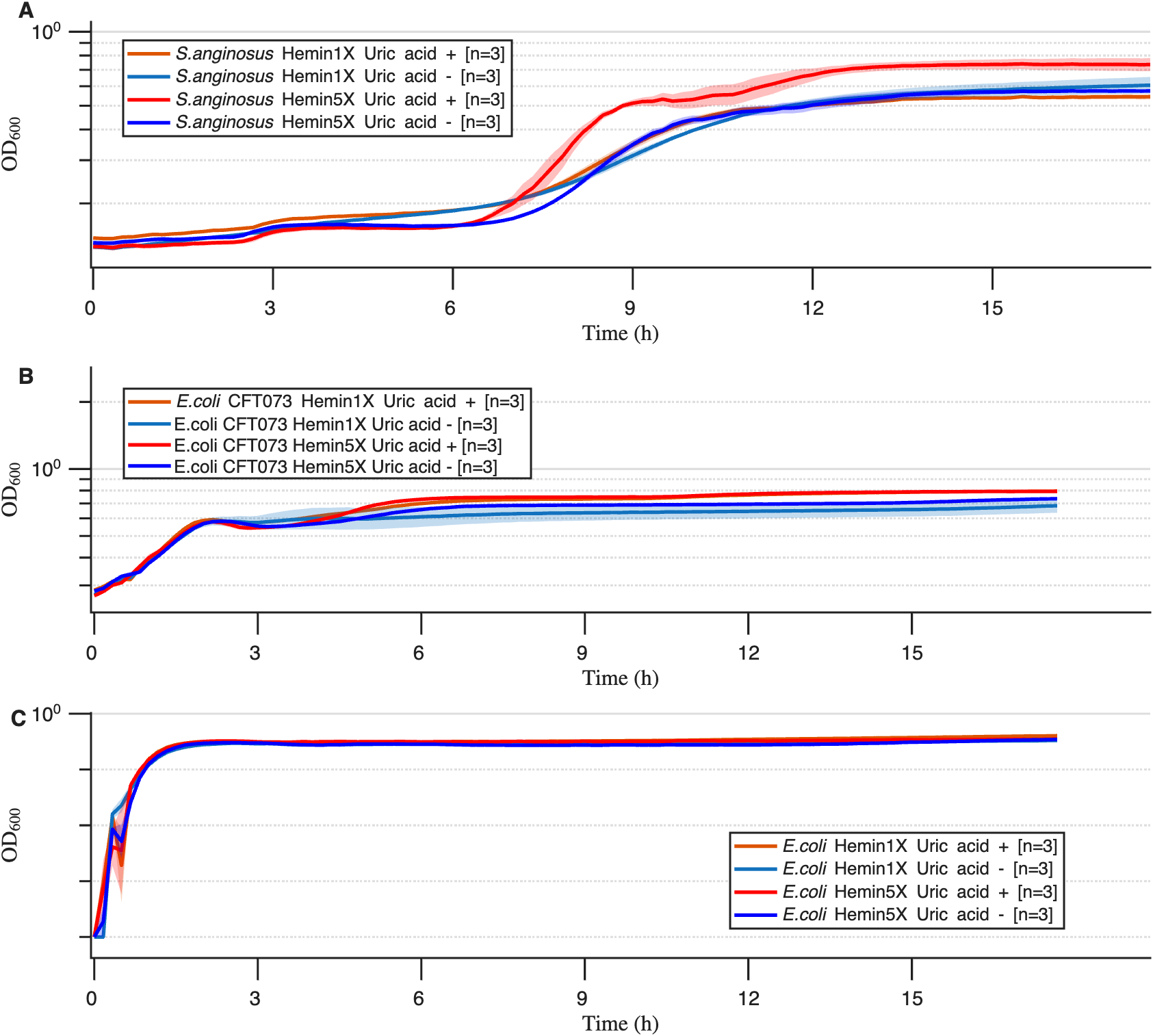


**Supplementary Figure 4:** Cultures of *S. anginosus* (UMB 3192) (A), *E. coli* (CFT073) (B) and *E. coli* (UMB3190) (C) in hemin-supplemented SimUrine.v2 in presence or absence of uric acid. Bold and light colors for high and low levels of hemin respectively, red tones for presence of uric acid and blue tones for absence. Growth (OD_600_) is shown on the y-axis; time (hours) on the x-axis. The formulation with 5X hemin and uric acid constitutes SimUrine.v3.

**Supplementary Table 1: Strains used on this publication**

| **Species** | **Reference** | **Tested to grow in SimUrine.v6 - 37°C** |
| --- | --- | --- |
| *Aerococcus urinae* | UMB5254 aka type strain ATCC 51268 | Not Tested |
| *Corynebacterium riegelii* | UMB12267 | Tubes, 5% CO2. Curves provided only for SimUrine.v4 |
| *Enterococcus faecalis* | UMB3193 | Tubes, 5% CO2. Curves provided only for SimUrine.v4 |
| *Enterococcus faecalis* | ATCC 29212 | Tubes and duets - plates, 5% CO_2_. Growth curves provided. |
| *Enterococcus faecalis* | UMB7540 | Tubes and 96-well plates, 5% CO_2_. Growth curves provided. |
| *Escherichia coli* | UMB1180 | Not Tested |
| *Escherichia coli* | UMB3190, aka ATCC 10798 | Tubes and 96-well plates, 5% CO_2_. Growth curves provided. |
| *Escherichia coli* | ATCC 25922 | Tubes and duets - plates, 5% CO_2_. Growth curves provided. |
| *Escherichia coli* (UPEC20) | NCBI BioSample database. Accession SAMN041053058. National  Library of Medicine, National Center for Biotechnology Information (RRID: SCR_004854). | Tubes and 96-well plates, 5% CO_2_ |
| *Klebsiella pneumoniae* | UMB9987 | Tubes and 96-well plates, 5% CO_2_. |
| *Lactobacillus crispatus* | UMB244 | Tubes and 96-well plates, Anaerobic. CFU/mL determined |
| *Lactobacillus crispatus* | UMB576 | Tubes and 96-well plates, Anaerobic. CFU/mL determined |
| *Lactobacillus gasseri* | UMB8273 | Tubes and 96-well plates, Anaerobic. CFU/mL determined |
| *Lactobacillus gasseri* | UMB8059 | Tubes and 96-well plates, Anaerobic. CFU/mL determined |
| *Lactobacillus gasseri* | UMB245 | Tubes and 96-well plates, Anaerobic. CFU/mL determined |
| *Lactobacillus gasseri* | UMB246 | Tubes and 96-well plates, Anaerobic. CFU/mL determined |
| *Lactobacillus gasseri* | UMB5255 | Tubes and 96-well plates, Anaerobic. CFU/mL determined |
| *Lactobacillus jensenii* | UMB8651 | Only in tubes, 5 % CO_2_. No CFU/mL determined |
| *Proteus mirabilis* | UMB7310 | Tubes and 96-well plates, 5% CO_2_. |
| *Proteus mirabilis* (strain 83) | Local strain (Croxall et al., 2011) | Tubes and 96-well plates, 5% CO_2_. Growth curves provided. |
| *Streptococcus anginosus* | UMB8616 | Tubes and 96-well plates, 5% CO_2_. Growth curves provided. |
| *Streptococcus anginosus* | UMB3192 | Tubes and 96-well plates, 5% CO_2_. Growth curves provided. |


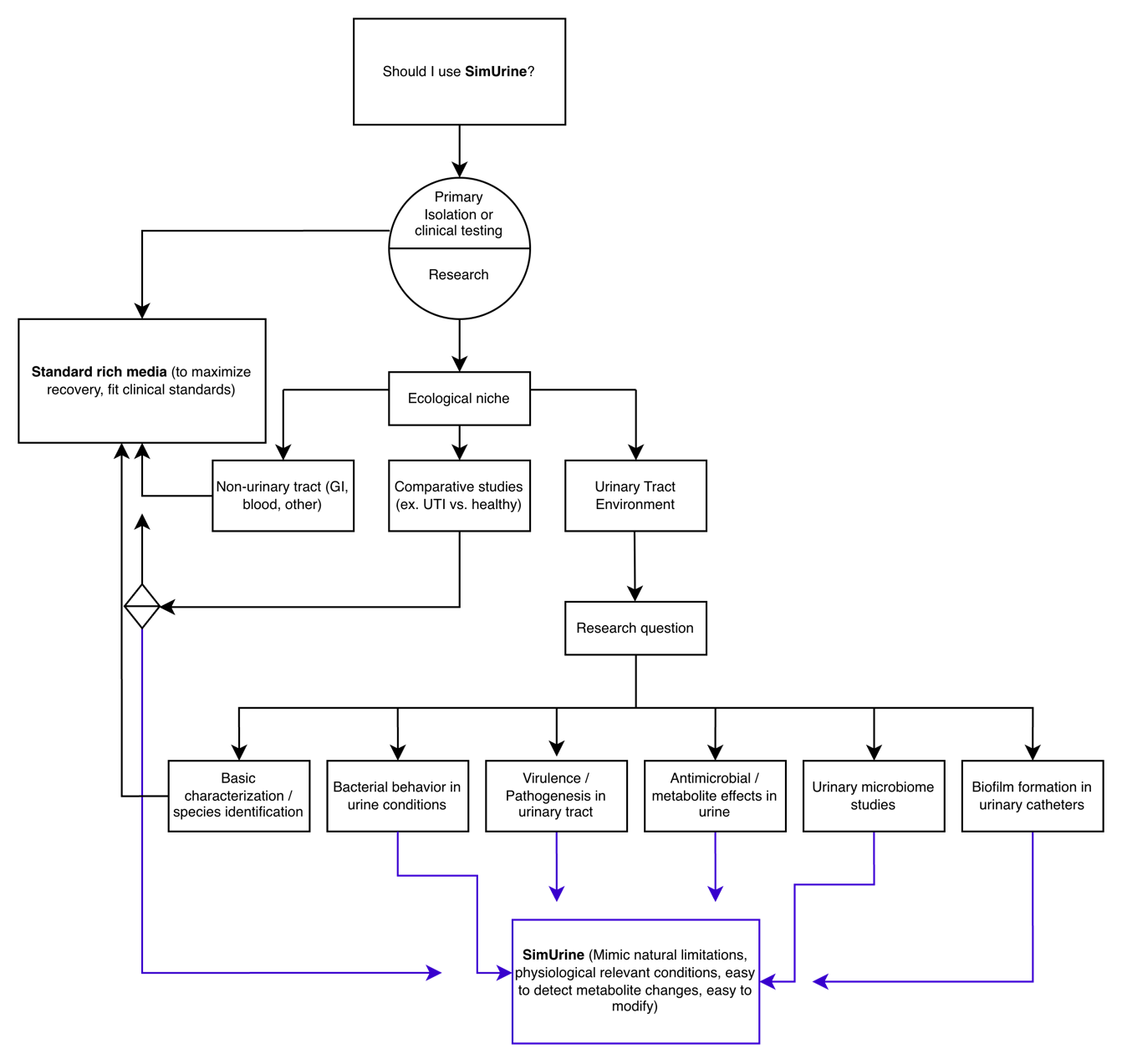


**Supplementary Figure 5: Decision tree on the potential use of SimUrine.** Arrows indicate directionality of the tree. Use of SimUrine is indicated in purple. Circle represents two independent processes; rhomboid indicates that both options might be valid or complementary.

**Supplementary Table 2: List of reagents utilized in this work.**

| **Correlative** | **Reagents** | **Code** | **Company** |
| --- | --- | --- | --- |
| 1 | Acetic acid | A6283-1L | Sigma-Aldrich |
| 2 | Alanine | 11442468 | Thermo scientific Chemicals |
| 3 | Arginine | A5006-100G | Sigma-Aldrich |
| 4 | Asparagine | A0884-25G | Sigma-Aldrich |
| 5 | Aspartate | A9256-100G | Sigma-Aldrich |
| 6 | Biotin | B4501-100MG | Sigma-Aldrich |
| 7 | CaCl_2_ | C1016-100G | Sigma-Aldrich |
| 8 | Citric acid | C0759-100G | Sigma-Aldrich |
| 9 | CoCl_2_*6H_2_O | 202185-25G | Sigma-Aldrich |
| 10 | Creatinine | 228940500 | Thermo scientific Chemicals |
| 11 | CuCl_2_ * 2H_2_O | 10588502 | Thermo scientific Chemicals |
| 12 | Cyanocobalamin | 1.2459201 | Sigma-Aldrich |
| 13 | Cystine | 11414814 | Thermo scientific Chemicals |
| 14 | FeCl_2_*4H_2_O | 10348150 | Thermo scientific Chemicals |
| 15 | Folic acid | F7876-10G | Sigma-Aldrich |
| 16 | Glutamate | 10502751 | Thermo scientific Chemicals |
| 17 | Glutamine | 11482277 | Thermo scientific Chemicals |
| 18 | Glycine | 1.00590.1000 | MERK |
| 19 | H_3_BO_3_ | B0394-100G | Sigma-Aldrich |
| 20 | Hemin | 10499291 | Thermo scientific Chemicals |
| 21 | HEPES 1M | H3375-25G | Sigma-Aldrich |
| 22 | Histidine | H8000-5G | Sigma-Aldrich |
| 23 | Isoleucine | I2752-1G | Sigma-Aldrich |
| 24 | KCl | 1.04936.1000 | MERK |
| 25 | L-cysteine | A10435.18 | Thermo scientific Chemicals |
| 26 | L-serine | S4500-1G | Sigma-Aldrich |
| 27 | L-threonine | 1.0841101 | Sigma-Aldrich |
| 28 | Lactic acid | 10665302 | Thermo scientific Chemicals |
| 29 | Leucine | L8000-25G | Sigma-Aldrich |
| 30 | Lysine | L5501-10MG | Sigma-Aldrich |
| 31 | Methionine | M9625-25G | Sigma-Aldrich |
| 32 | MgSO_4_*7H_2_O | 447155000 | Thermo scientific Chemicals |
| 33 | MnSO_4_*H_2_O | 11478237 | Thermo scientific Chemicals |
| 34 | n-acetyl-glucosamine | 11438770 | Thermo scientific Chemicals |
| 35 | Na_2_HPO_4_ | 1.06586.0500 | Sigma-Aldrich |
| 36 | Na_2_MoO_4_*2H_2_O | 10164830 | Thermo scientific Chemicals |
| 37 | Na_2_SO_4_ | 1.28211.1001 | Sigma-Aldrich |
| 38 | Na_2_WO_4_*2H_2_O | 10117423 | Thermo scientific Chemicals |
| 39 | NaCl | 207790010 | ACROS |
| 40 | NaH_2_PO_4_ | 1.06349.1000 | Sigma-Aldrich |
| 41 | NaOH | S5881-1K | Sigma-Aldrich |
| 42 | Na_2_SeO3 | 214485-5G | Sigma-Aldrich |
| 43 | NH_4_C_2_O_4_ | 10054013 | Thermo scientific Chemicals |
| 44 | NH_4_Cl | A4514-500G | Sigma-Aldrich |
| 45 | NH_4_SO_4_*7H_2_O | A5132-1K | Sigma-Aldrich |
| 46 | NiCl_2_*6H_2_O | 654507-5G | Sigma-Aldrich |
| 47 | Nicotinamide | 10447710 | Thermo scientific Chemicals |
| 48 | p-aminobenzoic acid | A9878-5G | Sigma-Aldrich |
| 49 | Pantothenic acid | 21210-5G-F | Sigma-Aldrich |
| 50 | Phenylalanine | 11468840 | Thermo scientific Chemicals |
| 51 | Proline | 11439512 | Thermo scientific Chemicals |
| 52 | pyridoxamine | 10785052 | Thermo scientific Chemicals |
| 53 | Pyruvic acid | 10562721 | Thermo scientific Chemicals |
| 54 | Riboflavin | 10593871 | Thermo scientific Chemicals |
| 55 | Serine | 11489113 | Thermo scientific Chemicals |
| 56 | Thiamine | 10477540 | Thermo scientific Chemicals |
| 57 | Tween80 | P4780-500ML | Sigma-Aldrich |
| 58 | Urea | U5378-1K | Sigma-Aldrich |
| 59 | Uric acid | U2625-100G | Sigma-Aldrich |
| 60 | ZnCl_2_ | 208086-5G | Sigma-Aldrich |
